# Envisioning the use of in-situ arm movement data in stroke rehabilitation: Stroke survivors’ and occupational therapists’ perspectives

**DOI:** 10.1371/journal.pone.0274142

**Published:** 2022-10-20

**Authors:** Hee-Tae Jung, Yoojung Kim, Juhyeon Lee, Sunghoon Ivan Lee, Eun Kyoung Choe

**Affiliations:** 1 Department of BioHealth Informatics, School of Informatics and Computing, Indiana University at IUPUI, Indianapolis, IN, United States of America; 2 Graduate School of Convergence Science and Technology, Seoul National University, Seoul, S. Korea; 3 College of Information and Computer Sciences, University of Massachusetts Amherst, Amherst, MA, United States of America; 4 College of Information Studies, University of Maryland at College Park, College Park, MD, United States of America; Duke Kunshan University, CHINA

## Abstract

**Background:**

The key for successful stroke upper-limb rehabilitation includes the personalization of therapeutic interventions based on patients’ functional ability and performance level. However, therapists often encounter challenges in supporting personalized rehabilitation due to the lack of information about how stroke survivors use their stroke-affected arm outside the clinic. Wearable technologies have been considered as an effective, objective solution to monitor patients’ arm use patterns in their naturalistic environments. However, these technologies have remained a proof of concept and have not been adopted as mainstream therapeutic products, and we lack understanding of how key stakeholders perceive the use of wearable technologies in their practice.

**Objective:**

We aim to understand how stroke survivors and therapists perceive and envision the use of wearable sensors and arm activity data in practical settings and how we could design a wearable-based performance monitoring system to better support the needs of the stakeholders.

**Methods:**

We conducted semi-structured interviews with four stroke survivors and 15 occupational therapists (OTs) based on real-world arm use data that we collected for contextualization. To situate our participants, we leveraged a pair of finger-worn accelerometers to collect stroke survivors’ arm use data in real-world settings, which we used to create study probes for stroke survivors and OTs, respectively. The interview data was analyzed using the thematic approach.

**Results:**

Our study unveiled a detailed account of (1) the receptiveness of stroke survivors and OTs for using wearable sensors in clinical practice, (2) OTs’ envisioned strategies to utilize patient-generated sensor data in the light of providing patients with personalized therapy programs, and (3) practical challenges and design considerations to address for the accelerated integration of wearable systems into their practice.

**Conclusions:**

These findings offer promising directions for the design of a wearable solution that supports OTs to develop individually-tailored therapy programs for stroke survivors to improve their affected arm use.

## Introduction

### Motivation

Stroke is a major cause of permanent motor impairments, affecting 800,000 people every year in the United States alone [1]. In the early stages of post-stroke—i.e., within a few months since the onset of stroke, also termed as the acute or subacute stages—survivors stay hospitalized and receive various rehabilitation therapies based on the characteristics and severity of their impairments [2]. Despite such efforts, approximately 65% of stroke survivors are discharged from hospitals with permanent motor impairments [3, 4]. Post-stroke motor impairments, particularly in the upper limbs, significantly deteriorate patients’ ability to execute essential activities of daily living [5] and their quality of life [6]. Hence, stroke survivors often need to continue rehabilitation therapies in the outpatient setting through their chronic stages (e.g., months or even years after stroke) to maximize their ability to perform functional activities and achieve independence in living [2].

The key to successful rehabilitation lies in the personalization of therapeutic strategies based on patients’ functional levels. In the conventional clinical setting, personalized treatments are often achieved based on clinical assessments of motor impairments using tools like the Fugl Meyer Assessment (FMA) [7] and Action Research Arm Test (ARAT) [8]. These tools are obtained based on trained clinicians’ observation of patients’ motor behaviors during predefined motor tasks within the clinic. However, prior studies have reported that improved motor function achieved and observed within the clinic (i.e., what patients are capable of doing, also termed as motor capacity) does not always lead to improvements in their functional ability within home and community settings (i.e., what patients are actually doing, also termed as motor performance) [9–12]. Considering that motor performance is what rehabilitation ultimately aims to improve, this gap between therapeutic goals vs. the lack of assessment tools for motor performance has been identified as a major barrier to developing optimal, personalized rehabilitation intervention strategies [13].

Over the past few decades, wearable technologies have received tremendous attention from clinical and research communities as an effective, objective tool to monitor patients’ motor performance in their naturalistic environments and evaluate rehabilitation outcomes to facilitate personalized care [13–17]. While the most widely adopted wearable form factor is the bilaterally-placed wrist-worn accelerometers, they also have been criticized for capturing primarily gross arm movements but not fine hand movements, which are of critical relevance to stroke rehabilitation [13, 18]. To counteract this limitation, more recently, finger-worn accelerometers have been actively investigated for their ability to capture both gross arm and fine hand movements [19–22]. With bilaterally-placed accelerometers (either wrist or finger-worn devices), a variety of measures can be derived from bilaterally-placed accelerometers to portray the comprehensive view of stroke survivors’ arm performance level [23]. The use ratio between the two arms has been the most widely accepted measure of arm performance [17]. First, the measure computes the use amount of each arm using different variables, such as use intensity (i.e., mean accelerometer magnitude) [13, 18, 24–26], time duration of active arm use (i.e., time duration when the accelerometer magnitude is greater than a certain threshold) [19, 27–29], and use variability (i.e., variation of accelerometer magnitude) [30, 31]. Then, a ratio between the measures obtained from the two arms is computed to quantify the relative use amount of the stroke-affected arm with respect to the unaffected arm. These ratio measures are often complemented by other measures, such as the absolute intensity and duration of active stroke-affected arm use, and whether and how often patients perform activities of daily living using the two arms simultaneously (namely, bilateral arm use) or only with the unaffected arm (namely, unilateral use) [23].

Yet, a wearable system capable of collecting a large volume of patient-generated data does not mean that it could be readily accepted by patients and therapists [32]. Prior studies have reported that sensing devices and patients’ arm movement data need to overcome a number of barriers before being successfully adopted and used in contemporary rehabilitation practice [33, 34]. Stroke survivors found it cumbersome to properly don and operate wearable sensors [33], and clinicians found that not all data support their therapy practice directly [17, 34]. In fact, despite decades of research, many—if not all—wearable solutions have remained a proof of concept and have not made their way into mainstream therapeutic programs [13, 17]. Translating and incorporating patient-generated arm performance data into everyday practice is not a trivial problem [19, 35–39]. Moreover, before all else, it is yet to be investigated if stroke survivors and therapists are willing to adopt wearable systems. Therefore, it is a critical prerequisite to bridge this gap through (1) understanding the receptiveness and use scenarios of the wearable technology and (2) investigating important design specifications to deliver practical values to its stakeholders: stroke survivors and therapists—particularly, occupational therapists (OTs) for the context of monitoring motor performance in naturalistic environments.

In this study, we elicit the perspectives of stroke patients and therapists on how sensor-based arm use measures could support contemporary rehabilitation practice. To obtain situated data representing stroke survivors’ real-world arm performance, we employed miniaturized finger-worn sensors placed on the index fingers in four stroke survivors over two consecutive days. We then used the collected arm use data to create study probes and performed semi-structured interviews with the stroke survivors and 15 therapists to elicit their opinions. Both patients and therapists perceived that accelerometer-based measures could support a unique opportunity to objectively monitor patients’ real-world arm performance. Particularly, therapists believed that wearable solutions could be seamlessly adopted in their contemporary service practice and support collaborative decision-making with patients to personalize therapy programs in and outside the clinical setting. Lastly, we further revealed design opportunities that can support the wider adoption of wearable solutions in the rehabilitation practice.

### Related work

The emergence of patient-generated health data—including technology-based and patients’ self-reported data—has been reshaping how patients engage in the care practice and how clinicians provide care [40, 41]. These data collected outside the clinic setting could reveal profound insights about patients’ day-to-day behaviors [42] and empower stakeholders in health care: clinicians can use the data for diagnosis, treatment, and remote monitoring [43–45], patients and clinicians can make data-driven decisions collaboratively [40, 46, 47], and patients can take an active role in managing their health and well-being [48, 49].

Similarly, an increasing volume of research efforts is made to support stroke rehabilitation in the outpatient setting using patient-generated health data. Many studies have proposed various research prototypes to monitor if and how stroke survivors adhere to at-home therapeutic exercises using different technologies, such as wearable sensors [36, 38, 50, 51], video cameras [52], and rehabilitation games [53, 54]. These systems can potentially provide therapists with valuable information, such as stroke survivors’ motor capacity (e.g., range of motion [52, 55]), engagement with therapy (e.g., frequency and duration of participation in training) [56–58], and their subjective experience during at-home therapeutic exercises [59]. This could, in turn, be used to better personalize patients’ at-home exercise programs. However, survivors’ arm capacity data during relatively short therapeutic exercise periods (e.g., $\tilde{3}0$ minutes) does not illustrate their general arm usage in daily living, a topic of research that remains relatively underexplored. Ploderer et al. have investigated how stroke survivors’ arm use amount data could be visualized (i.e., dashboard in the authors’ term) and reported that OTs found the shared data useful [34]. However, the authors primarily focused on investigating the optimal information visualization for therapists to understand the clinical insights from wearable data rather than how the data could be situated and used in real-world practice and support personalized therapy programs.

While we share similar goals to these prior studies in understanding whether and how patient-generated sensor data could facilitate data-driven decision-making through patient-provider collaboration, we extend this line of work in two ways. First, we examine opportunities to utilize sensor data in outpatient occupational therapies, a novel context where sensor-based, objective measures of arm performance could have great value for both patients and therapists, as we demonstrate in this study. We investigate how patient-generated data should be situated in a specific clinical context while minimally disrupting the current workflow for both patients and therapists. Second, we introduce an unfamiliar stream of health data—a stroke survivor’s everyday arm performance data collected from a pair of finger-worn ring sensors—to stroke survivors and therapists. We examine stroke survivors’ and therapists’ receptiveness toward the data and how they make sense and use of the data.

## Methods

**Fig 1:**
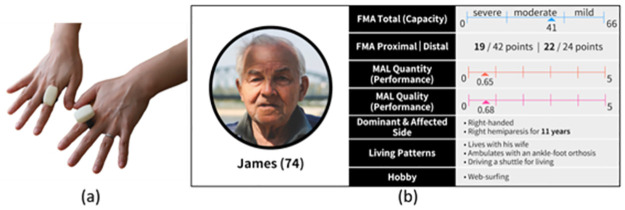
(a) The picture of our finger-worn sensors on the index fingers. (b) The patient profile was created using the data we collected with an actual stroke survivor (i.e., P2) in his chronic stage. The full motor capability of a patient in the clinical setting is assessed using the Fugl-Meyer Assessment, which is one of the most widely used tools in the clinical setting worldwide [60]. The Fugl-Meyer Assessment contains the proximal portion (i.e., shoulder and elbow) and the distal portion (i.e., hand and finger movements). The patient’s scores suggest that he has relatively limited shoulder and elbow motor capacity (19 out of 42 points) while he has a decent distal motor capacity (nearly perfect scores: 22 out of 24 points). The motor performance perceived by patients themselves during their daily living is provided in the Motor Activity Log (MAL)the Motor Activity Log (MAL), which is another standardized assessment tool based on patients’ self-reports [61]. The quality and the quantity of his perceived arm performance are scored separately. His Motor Activity Log scores suggest that he does not use his stroke-affected arm when executing activities of daily living (0.65 out of 5), and the quality of movement does not contribute to completing the activities (0.68). He drives a shuttle for a living.

**Fig 2:**
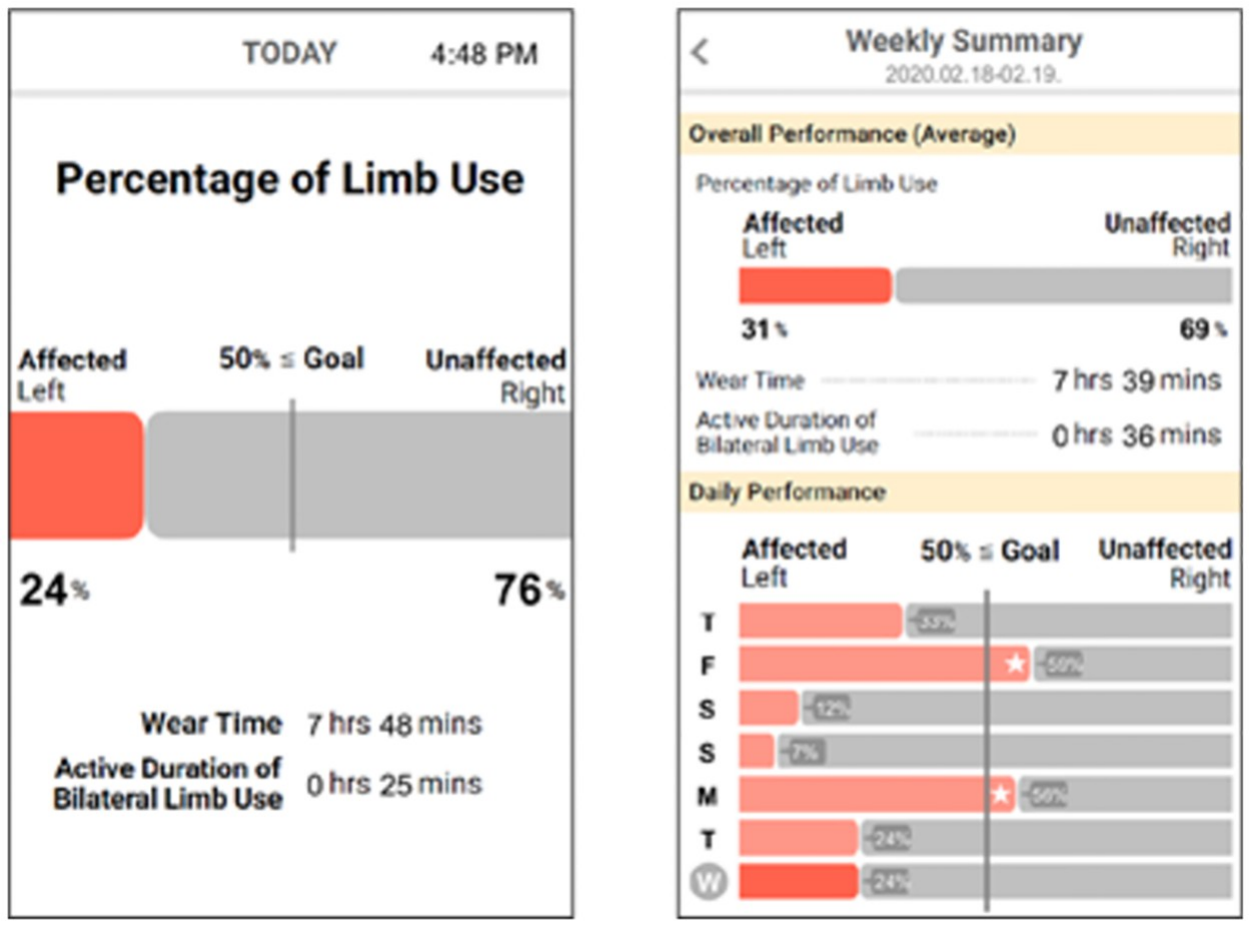
One of the study probes—a basic bar chart—that we designed and presented to P1 based on her own data: daily performance (left) and weekly performance trajectory (right). The patients’ response to the chart design is beyond the scope of this study, and hence the rest charts are omitted in this paper.

**Fig 3:**
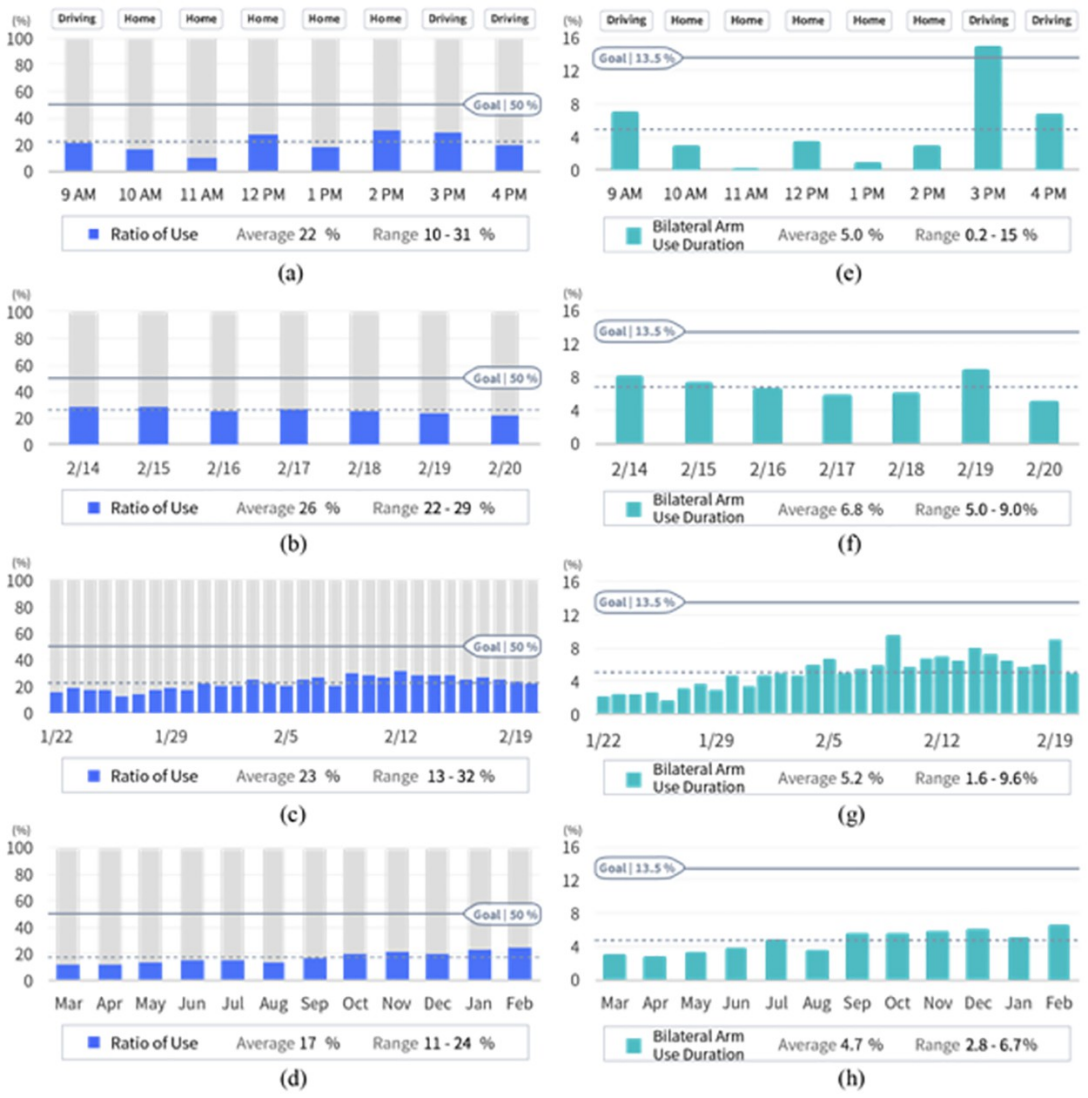
Example bar charts that we presented to OTs: Ratio of Use (left) and Bilateral Arm Use Duration (right). The data shown in the charts for Affected Arm Use Duration are similar to those in the Bilateral Arm Use Duration charts and hence are omitted here due to space limitation. The day graphs, (a) and (e), presented the averaged value of the measures for each hour, which were obtained from one of the two days (i.e., the second day) data that we collected with James. We showed the average, minimum, and maximum values of the measure across the eight-hour period at the bottom of the graph. The average value was also presented on the bar graph using a dotted gray horizontal line. The solid line represented the goal that therapists could set for a particular patient to reach, although we arbitrarily set the goal (e.g., 50% for the Ratio of Use that represents balanced bilateral arm use) for the presentation purpose. The annotations shown above the bars (e.g., Driving and Home) were computed using the Google Maps Timeline data obtained from the smartphone that was given to James. The week, month, and year graphs similarly presented the three performance measures. Because we only collected data from James over two days, all data, except for the data pertaining to the last two days of the week graph (i.e., data on 2/19 and 2/20 in Figure(b) and (f)), were synthesized to represent some plausible trends of arm performance level (e.g., improvement or fluctuation).

We conducted semi-structured interviews with four stroke survivors in their chronic stage and 15 OTs; all 19 participants completed the study. The goal of the interviews was to gain insights into (1) stroke survivors’ and OTs’ willingness or barriers to accepting the concept of tracking and using in-situ arm movement data, (2) how stroke survivors and OTs envision leveraging patient-generated arm movement data to personalize therapy programs to increase the survivors’ affected arm use, and (3) how a sensor-based system can be designed to meet the needs of stakeholders and translated into practice. To this end, it was important to situate stroke survivors and OTs in a realistic and concrete scenario (e.g., type of data that can be collected) while giving them enough room to envision new ideas. Hence, we first deployed a pair of finger-worn sensors to the stroke survivors in their routine daily living for two consecutive days to collect arm use data (see Fig 1A). We then computed clinically validated measures of arm use, created study probes for stroke survivors (Fig 2) and OTs (Fig 3), and conducted semi-structured interviews to elicit their opinions. In the following, we describe study participants, study probes along with data collection, and analysis methods in detail. None of the participants had prior relationships with the authors. The study procedure was reviewed and approved by the Institutional Review Board (IRB) of the University of Massachusetts Amherst. All participants provided written consent that described the benefits and risks of this research project prior to the interview. The study procedure was reviewed and approved by the Institutional Review Board (IRB) of the University of Massachusetts Amherst. All participants provided written consent that described the benefits and risks of this research project prior to the interview.

### Participants

#### Stroke survivors

Stroke survivors were recruited from November 2019 until March 2020. The recruitment was conducted through fliers in the stroke support groups, community-based rehabilitation programs, and nursing homes. The inclusion criteria for stroke survivors required that they were (1) 18 years old or older, (2) in their chronic stage (i.e., one year or longer since their latest stroke), had (3) mild upper-limb motor impairment (i.e., 40 points or greater in FMA), and (4) close-to-normal or normal cognitive function (i.e., 24 points or greater in Mini-Mental State Examination; MMSE). The cutoff for FMA was selected to screen stroke survivors with small amount of movements that are deemed difficult to benefit from using wearable sensor. The cutoff for MMSE was selected to ensure that the participating stroke survivors can understand the study probes and explain their thoughts and opinions. The cutoff for FMA was selected to screen stroke survivors with small amount of movements that are deemed difficult to benefit from using wearable sensor. The cutoff for MMSE was selected to ensure that the participating stroke survivors can understand the study probes and explain their thoughts and opinions. Participants were screened for eligibility via phone calls. Table 1 shows the demographic and clinical information of the stroke survivor participants.

**Table 1:** The demographic and clinical information of the stroke survivors.

ID	Gender	Age	Chronicity	FMA	MAL		Education	Occupation
					Quantity	Quality		
*P*1	F	63	4.1	58	3.16	2.83	Post Graduate	Retired
*P*2	M	74	11.3	41	0.65	0.68	Some College	Self-employed
*P*3	F	66	11.2	42	1.31	1.35	Some College	Retired
*P*4	M	75	3.3	63	5	3.9	High School Graduate	Full time

#### Occupational therapists

The recruitment process for OTs started in July 2020. The recruitment was conducted through words of mouth, email fliers, and online advertisement (i.e., www.aotf.org). The inclusion criteria for the OTs required that they (1) were 18 years old or older and (2) had more than three years of experience as an OT at the time of recruitment. Participants were screened for eligibility via emails. We completed the data collection with 15 OTs by September 2020, at which all the authors agreed that data saturation was established. Table 2 shows the demographic information of the OT participants.

**Table 2:** The demographic information of the OTs.

ID	Gender	Location (State)	Practice years	Current position	Practice setting
*T*1	F	MA	10	Graduate student	Outpatient
*T*2	F	MA	5	Graduate student	Inpatient, Outpatient
*T*3	F	GA	6	OT	Outpatient
*T*4	F	GA	4	Professor	Inpatient, Outpatient
*T*5	F	MA	11	OT	Inpatient
*T*6	F	MA	20	OT	Inpatient, In-home care
*T*7	F	IL	17	OT	Outpatient
*T*8	F	NJ	14	Professor	Inpatient
*T*9	F	TX	5	OT	Inpatient
*T*10	F	NJ	7.5	OT	Inpatient, Outpatient
*T*11	F	MA	24	OT	In-home care, Inpatient, Outpatient
*T*12	F	OH	11	OT	Outpatient
*T*13	F	TX	6	OT	Outpatient, Inpatient
*T*14	M	DC	8	Postdoctoral researcher	Outpatient
*T*15	F	MD	6.5	OT	Outpatient

### Study procedure

#### Stroke survivors

The goal of the study with stroke survivors was twofold: (1) to investigate stroke survivors’ perspectives and experiences on wearing finger-worn sensors in their daily living and (2) to collect sensor-generated data to create study probes for the stroke survivors as well as OTs. The eligible study participants were assessed for their cognitive capacity (measured by Mini-Mental State Examination), motor capacity (measured by Fugl-Meyer Assessment), and motor performance level (measured by Motor Activity Log) by trained research staff. After completion of the assessments, we deployed finger-worn sensors to the four recruited chronic stroke survivors on their index fingers bilaterally, as shown in Fig 1A. Participants wore the sensors for eight hours a day over two consecutive days, starting and ending at their convenience, mostly depending on the beginning time of their waking hours. We provided a GPS-enabled smartphone (Samsung Galaxy A20) pre-installed with Google Maps app to derive contextual information related to their locations (e.g., whether stroke survivors were at home or driving). Upon completion of the sensor data collection and before the interview, the research team created study probes based on the ratio measure (Fig 2), which summarized the amount of the affected arm use with respect to the contralateral arm (denoted as Ratio of Use hereafter)(denoted as Ratio of Use hereafter) [26, 28]. Given the stroke survivors’ cognitive impairment, albeit mild, we only presented the Ratio of Use measure among other sensor-based measures because it would be the easiest to comprehend. We conducted an hour-long in-person interview the next day of the data collection at their preferred time and location (i.e., three at their home and one in the meeting room on campus). The research team first explained the goal of the study, presented them with the visualized study probe, explained how to interpret the information, and asked about their willingness to wear the sensors in their routine daily living and how to utilize the sensor-generated data to induce a greater level of affected arm use during their daily living.

#### Occupational therapists

To investigate OTs’ perspectives on how to use patient-generated sensor data to personalize therapy programs, we prepared the study probe based on the arm performance data that were collected from the stroke survivors. Although our initial intention was to share all four stroke survivors’ data with OTs during the interview, in the interest of time, we selected one of the four stroke survivors after a careful review of the data and clinical information; P2 demonstrated a significantly low performance level (measured by the Motor Activity Log [25]) despite a relatively high capacity level (measured by the Fugl-Meyer Assessment [7]), as shown in Table 1 and Fig 1. We selected this stroke survivor to elicit OTs’ perspectives on how patient-generated sensor data could complement their practice towards maximizing stroke survivor’s performance. Based on a pilot study with two therapist volunteers, we designed the study probe to include two components: (1) the clinical and demographic profile of the pseudonymized stroke survivor, James (see Fig 1B), and (2) study probes for the three types of performance measures derived from the collected sensor data (see Fig 3). We expected that this information, along with a fabricated image of James, could provide OTs with a realistic and concrete therapeutic scenario to help them envision how to leverage sensor data as part of their real-world clinical practice.

We introduced three representative measures of arm performance that were derived from James’ sensor data as we believed OTs would be capable of interpreting detailed sensor-based information. The measures include (1) Ratio of UseThe measures include (1) Ratio of Use [26, 28], (2) the duration of affected arm use, denoted as Affected Arm Use Duration, (2) the duration of affected arm use, denoted as Affected Arm Use Duration [18, 29], and (3) the duration of bilateral arm use, denoted as Bilateral Arm Use Duration, and (3) the duration of bilateral arm use, denoted as Bilateral Arm Use Duration [19]. For each measure, we provided bar graphs with four different time granularities (i.e., day, week, month, year), as shown in Fig 3. The study probe was iteratively revised through the pilot interviews with two therapist volunteers. The data that we collected from the pilot interviews were not included for further analysis.

Interviews were conducted via an online conferencing platform (i.e., Zoom) at their preferred time for approximately one and half hours. The research team first explained the goal of the study and asked participants about their routine clinical workflow and how they evaluate stroke survivors’ capacity and/or performance. Then, the research team presented the clinical and demographic information of the representative stroke survivor (Fig 1), followed by the study probes and detailed explanations of the three sensor-based measures of arm use (Fig 3). We asked the study participants about their perceived usefulness of the presented data. We also asked about specific ways that participants would use the data to set therapy goals and personalize therapy programs for individual patients (e.g., How would you use the sensor data when setting arm use goals for individual patients?). Last, we asked study participants about the potential effects of using sensor-based patients’ performance data on the quality of their therapy practice (e.g., How would the sensor data affect your therapy practice?). The first author participated in all fifteen interviews, and the rest of the authors participated in interviews based on their availability. For each interview, no more than three authors participated to avoid overwhelming the study participants with many interviewers and to construct an amicable and engaging online interview environment.

### Analysis

All interviews were audio-recorded, which were then transcribed and analyzed using the thematic approach [62], using ATLAS.ti Cloud. At first, three authors (HJ, JL, YK) individually analyzed the transcriptions using open coding. Then, all authors contributed to the affinity sessions to build a coding scheme (i.e., axial coding). After iterative processes of open coding and discussion, all authors reached the saturation of codes and agreed on the coding scheme. The final coding scheme included three main codes and 11 sub-codes (see S2 File). Three authors coded the rest of the interview data according to the coding scheme. Then, all the authors reviewed the codes and developed the themes in an iterative manner until all authors came to an agreement on the themes. The detailed background of the authors is provided in S1 File.

## Results

In this section, we present our findings focusing on (1) the receptiveness of using wearable (i.e., finger-worn) sensors and the in-situ arm movement data by stroke survivors and OTs, (2) opportunities of using the patient-generated sensor data to personalize therapy programs, and (3) potential ways to improve the remote monitoring system to further support its translation into the contemporary therapy practice.

### Receptiveness of the ring sensors and arm movement data

Both stroke survivors and OTs were generally positive toward the use of sensor-generated arm movement data in their routine practice to enhance the quantity and quality of rehabilitation service. They shared the situations in which the sensor data would be helpful or not and the rationale.

#### Willingness to wear the ring sensor by stroke survivors

Three out of four interviewed stroke survivors (P1, P2, P3) expressed their willingness to wear the proposed ring sensors in their daily living for a prolonged time conditioned on the endorsement by their clinicians and the perceived therapeutic benefit of using the sensors. When they were asked how long they would be willing to wear the sensors, for instance, P1 answered “I would be willing to wear it if I needed to. Three weeks or in front of the appointment or whatever.” Stroke survivors explained that they would want to use the sensor data to monitor their own performance, receive social support from people in close relationships (e.g., family members), and share the data with OTs for rehabilitation therapies of greater quality. On the other hand, P4 reported that he would not use the proposed finger-worn sensors because he did not believe that accelerometer-based sensors could address the needs he felt—reducing pain. Despite his close-to-normal motor capacity (63 out of 66 points in FMA) and performance (5 out of 5 points in MAL Quantity) as shown in Table 1, P4 was suffering from chronic pain on his affected arm. He stated that he would not wear the sensor unless it can address the pain he suffered.

P3 was particularly interested in using the sensor data to justify financial subsidy from third-party payers to receive more rehabilitation therapy sessions. Because rehabilitation therapy is particularly costly in the US, they were not able to receive as much therapy as they wanted unless the therapy-related expenses were supported by third-party payers. For instance, P3 explained that she was experiencing challenges incorporating fine-hand movements (i.e., using her fingers) in her daily living and wanted to receive more therapy. However, due to the lack of objective means to monitor her progress in fine-hand movements, she was not able to receive enough subsidy from the third-party payers for rehabilitation therapies. P3 stated “*You always had to prove that you can make progress [to receive third-party support]*.”She stated that the sensor data could serve as objective evidence, demonstrating the progress she could make in her daily living.

Stroke survivors did not feel ashamed of or discouraged from wearing the sensors in public. Unlike consumer-grade ring sensors such as Oura ring [63] or Amazon Echo Loop [64], the deployed finger-worn sensors were unwieldy (Fig 1A) and were easily noticeable by other people. During the data collection, stroke survivors engaged in their natural daily activities, such as going to public libraries (P1), banks (P2), and restaurants (P3), and were asked by others about the sensors they were wearing. For instance, P2 shared his experience:

*I went to the bank to cash a check, … the woman says, “What is that?” So I had an opportunity to explain briefly what it was*.

When we asked him if such attention made him feel awkward and discouraged him from wearing the sensors, “I couldn’t care less”

#### Incorporation of the sensor data in the rehabilitation practice by OTs

OTs were willing to adopt the sensor data in their routine rehabilitation practice because they found using the sensor data would be minimally burdensome while useful to provide more patient-specific rehabilitation therapies. OTs believed that collecting and reviewing the patient-generated movement data impose a negligible extra workload compared to the conventional capacity-based assessment tools where OTs must spend 30–45 minutes to administer. T8 stated:

*I think it’s information […] is not burdensome to collect. It’s automated information that comes in, that can be populated. So, I feel like the burden of collection is low*.

OTs explained that, given the collected data, they would first review the data “*in the morning or right before seeing the patient*” (T5) for a few minutes to get an overall sense of patients’ daily performance patterns. Then, OTs would spend more time “*reviewing [the data] with the patient during the session*” (T1) and have more in-depth discussions with their patients during in-person therapy sessions to construct therapy goals around patients’ daily performance and personalize therapy programs. We asked OTs if the collaborative decision-making process informed by the patients’ sensor data would cause additional challenges, such as more confined time management during in-person therapy sessions. OTs answered that obtaining detailed contextual information about patients’ daily living and their arm use is already an essential part of their workflow and thus would not disrupt their clinical routines. T10 explained:

*I don’t really see how [using the sensor data] would be extra work because [… the sensors] are giving us more information. We’re already asking those questions like, […] “How long did you use [your affected arm] for? Why was [using your affected arm] difficult? Why didn’t you use [your affected arm] during this [time]” And a lot of times we’re getting the collateral information from the caregivers and the family members. So, I don’t think that it’s creating any extra work*.

Our analysis suggests that OTs’ positive attitude towards using the sensor data is, in part, due to the easy access to computers and their significant exposure to digital monitoring devices in their contemporary rehabilitation practice. All the OTs we interviewed explained that they have access to computing devices, such as desktop or laptop computers, in their current work environments. Many of them said they carry laptop computers when they see patients during in-person therapy sessions. Some OTs stated that, in addition to computers, they were actively using other digital devices in their in-person sessions. For instance, five OTs (T5–T7, T12, T13) stated that they routinely use a digital dynamometer (i.e., a device that measures patients’ grip strength). T13 reported that, in her routine practice, she used a touchscreen-based solution that supports in-person motor assessment and rehabilitation exercises, namely Bioness Integrated Therapy System. In addition, when we first introduced the ring sensors during the interview, OTs were able to relate the ring sensor to Fitbit and, more broadly, accelerometer sensors. Thanks to such exposures, OTs felt comfortable adopting a new sensor-based solution in their practice and accessing the data using computing devices. When asked for the preferred form of computing devices, many answered that they would want to use either a laptop or a tablet computer for portability and familiarity.

### Opportunities to support performance-driven therapy personalization

OTs regarded that patient-generated daily performance data have great potential to support patients’ self-management and motivate patients to use their affected arm use in their daily living more frequently by devising patient-centered rehabilitation therapies. More specifically, OTs envisioned (1) setting goals around daily performance, (2) enabling collaborative customization of therapy programs for individual patients, and (3) personalizing the frequency and the form of therapy sessions.

#### Personalized therapy goals around daily performance

Our analysis supports that the sensor data can help OTs define quantifiable goals throughout the therapeutic programs in the context of patients’ real-world performance, which better reflects patients’ ultimate goals for therapy that are often related to daily functioning. As such patient-centered therapy goals could motivate patients to use their affected arm more in their daily living, OTs were willing to incorporate the sensor data in their clinical practice. For instance, T15 stated:

*So, say James were to come to me tomorrow, and I could look at this data and review it with him. [… and] use this information to write goals and to motivate patients*.

When setting therapy goals, OTs would use the sensor data of different granularity and duration to reflect various factors, such as patients’ living patterns, health conditions, and impairment level, that may collectively affect their daily performance. For example, T13 exemplified a scenario in which the sensor-based measures could be used to set therapy goals on a daily or weekly basis while specifically considering the patient’s unique living and health conditions:

*[If a patient says] I’m tired in the afternoons. [I would write a therapy goal as] the patient will demonstrate increased use of the right upper extremity during functional activities from the hours of 9:00 AM to 12:00 PM as measured by [… the] ring sensor. [For example,] an improvement from 22% average to 30% average on five or seven days out of the week. I think that would absolutely be a measurable therapeutic goal*.

OTs would selectively or collectively use different sensor-based measures (i.e., Ratio of Use, Affected Arm Use Duration, and Bilateral Arm Use Duration) to write more personalized therapy goals to accommodate stroke survivors’ different impairment levels. For instance, for patients with mild motor impairments, OTs would use the Affected Arm Use Duration measure to induce a greater level of the affected arm use, as T1 explained: “*for someone who […] are considered mild, […] the goal is … they use the affected arm … [for] 90% of the waking hours*.” On the other hand, for patients with more severe motor impairments and poor motivation to use their affected arm, OTs would use Bilateral Arm Use Duration to write goals to encourage patients to start using their affected arm by assisting with their unaffected arm, as T10 explained:

*For some patients, the bimanual stuff is really important, especially if they’re inattentive. “Don’t just use the single hand, make sure you’re using the other hand together.” Sometimes that’s an easier place to start than just forcing them to use the affected side*.

When setting personalized therapy goals, OTs would primarily reference patients’ own performance levels as a benchmark. More specifically, OTs would review patients’ prior performance and define short-term and long-term goals to make sure that patients stay motivated and continue to use their affected arm, as T14 stated:

*Because part of setting a goal in therapy is not to make it too difficult […] if he can only do 24% right now and you ask him to do 70%, [then] it may seem too difficult, too unobtainable. So perhaps 50% could be a short-term goal, with 70% being a long-term goal*.

Then, during follow-up visits, OTs would review patients’ performance level since the last clinical visit—collaboratively with patients—and incrementally adjust the performance goal. T13 suggested an example scenario while reviewing Fig 3(b):

*Together with the patient, you could collaboratively identify, [while referencing the example in Fig 3] “Okay, on average, you’re using your right upper extremity […] approximately 22%, […] do you think it would be reasonable this next week to try to shoot for 30%?” […] You collaborate with the patient [to] make that goal together*.

OTs envisioned that such collaborative efforts to set realistic, measurable goals based on patient-generated sensor data could induce a greater level of arm use in patients’ daily living.

While OTs generally found the performance data presented in Fig 3 useful and relevant, some expressed the need for additional information to set therapy goals more effectively. Three OTs (T7, T11, T13) wanted to have access to normative data—for example, from a larger population of stroke survivors with similar motor impairments or age-matched healthy individuals—when setting therapy goals. Due to the lack of normative data, despite the usefulness of the wearable sensor as a performance assessment tool, one therapist was cautious about defining therapy goals based on the sensor data as it may counter the principle of evidence-based practice (T8). Furthermore, OTs identified that the presented sensor-based performance measures primarily capture the amount of arm use without necessarily reflecting the quality of the performed movements (e.g., the presence of compensatory movements, such as leaning forward with the body to reach for a target rather than using the arm muscles). Consequently, OTs would concurrently reference some existing measures related to movement quality, such as patients’ range of motion or severity of muscle spasticity, to get a more comprehensive view of the patient’s motor condition when setting therapy goals.

#### Collaborative personalization of therapy programs

All the OT participants supported that sensor-based performance measures could enable OTs to better collaborate with their patients to devise more personalized strategies for individual patients to engage the affected arm in their daily context. More specifically, the sensor data could help OTs obtain an in-depth understanding of their patients’ living patterns and daily motor performance, which would lead to better personalization of therapy programs. T11 explained:

*This [sensor data] prompts a whole bunch of different questions. You can learn more about what they’re doing, when they use their arm, and when they don’t use their arm, and it can lead to much deeper conversations about ‘why is it hard to use your arm when you’re doing that particular task?’ I mean, it could lead your interview [with your patient] to a much deeper level*.

During the interviews, OTs showed interest in portions of the sensor data with distinctive patterns, such as the peaks and troughs (e.g., at 3 PM and 11 AM in Fig 3(e), respectively). Those data points may offer opportunities to learn the underlying factors that have resulted in different levels of the affected arm use. For instance, OTs would ask patients about the activities that patients were engaged in at the peaks and encourage the patients to perform such activities more frequently, as T11 said while looking at Fig 3(a):

*I would want to know what [the patient] was doing at 12 o’clock, 2 PM, and 3 PM […] because that’s when [the patient] used his arm the most. […] Then I would try to tap into whatever [activity] that was happening, to see if we could do more of that [activity]*.

Similarly, OTs would investigate the activities that patients were engaged in at troughs and collaboratively devise strategies to increase the affected arm use at those times. T8 explained, “*What was specific to that activity that he, for whatever reason, didn’t use the affected arm as much? Is there something else we can figure out, like a role for the affected arm [in the activity]*” Some suggested that troughs could indicate the time when patients were sedentary during the day and potentially be a good time to perform the prescribed at-home exercises. For instance, when T13 spotted that James did not actively use his affected arm at 11AM in Fig 3(a), she conjectured if James was demonstrating a habitual sedentary behavior (e.g., watching his favorite TV show) and wondered if she could prescribe home exercises at that time:

*Well, to help him achieve his goal of getting more active, maybe we work on like 1–2 minutes of exercises he could do [during] every commercial break. He’s going to do one of these three exercises*.

While OTs appreciated seeing the auto-generated annotations from smartphone-based GPS data (Fig 3(a) and 3(e)), some expressed the need for more fine-grained contextual information that can better explain the activities patients were engaged in at different times. Such annotations, along with the sensor data, could provide a more in-depth understanding of the kind of activities to promote or discourage. As one of the means to obtain such information, some suggested the idea of providing patients with a mobile app to self-annotate their activities, as T12 suggested:

*They could go to their little tablet during the day […] and can choose from a cleaning task or a feeding task or whatever. That way, we can see exactly what they were doing at those times*.

#### Personalization of the frequency and form of therapy sessions

OTs envisioned having in-person therapy sessions flexibly based on the patient-generated sensor data. In the current rehabilitation practice in the U.S., the number of rehabilitation therapies is mostly dictated by the amount of financial subsidy patients can receive from third-party payers. More specifically, most third-party payers have a limit for the rehabilitation services that patients can have per year, either in terms of the number of in-person therapy sessions or monetary support that covers the in-person sessions. For instance, T12 explained that “*[Medicare] gives us up to a rough $3,700 for therapy services [in a single year], and we could maybe get 40 sessions out of that*.” Subsequently, the number of therapy sessions and the total duration of the overall rehabilitation service that stroke survivors can receive are limited. As OTs would be able to monitor patients’ daily performance using the sensor data, OTs anticipated having in-person therapy sessions less frequently than what they would do in the conventional rehabilitation practice. OTs explained that they could extend the total duration of the overall rehabilitation service by either reducing the frequency of in-person therapy sessions or opportunistically determine when patients should come in for an in-person session, which in turn will maximize patients’ benefits from third-party coverage. For instance, T7 explained:

*I might see [the patient] two times a week for the first two weeks to get [the patient] set up on this […] then I might drop [the frequency of in-person sessions] to every other week, […] because I am going to have a bigger chunk of data coming in still*.

Furthermore, some stated that they might consider having more remote therapy sessions or brief follow-up sessions via videoconferencing, which may further reduce the associated medical costs. Since a great portion of rehabilitation therapies focuses on “*making modifications to the exercise plan*” or “*educating them about the exercise plan they’re supposed to be doing*”, some OTs stated that they would “*opt to do more virtual sessions*” (T14). OTs believed that virtual therapy sessions, in combination with in-person therapy sessions, would “*prolong […] patients’ insurance benefits […] that actually maximizes the efficiency of the system and provides continued care*” (T8).

### From promise to delivery: Practical challenges and design considerations

Our analysis surfaced substantive issues that need to be reflected in the wearable system to accelerate its adoption in real-world settings, which are centered around (1) the sensors’ form factor, (2) variability in stroke survivors’ technology proficiency and preference, and (3) OTs’ important roles to incorporate sensor data in clinical practice.

#### Sensor-wearing experience

Both stroke survivors and OTs reported issues related to the sensor form factor, but their focus was different. The four stroke survivor participants shared their sensor-wearing experience based on the two-day data collection; hence their comments were related to day-to-day affairs and comfort. On the other hand, OTs considered how a broader range of impairments (e.g., limited motor skills and edema) might affect the sensor-wearing experience.

According to stroke survivors, the point of discomfort was mainly caused by the size and texture of the ring sensor. They said that the current size of the finger-worn sensors made it difficult to don and doff clothes or gloves (P3, P4) and put their hands into the pocket (P3). Furthermore, P2 reported that friction caused by the material of the ring sensor hindered his arm from moving against the object as he pointed out, “*I didn’t like that when I put my hand [with the ring sensor] on something it just stopped dead. It’s sticky*.” P2 and P3 further explained that such experience could make them uncomfortable when using their hands in daily activities. For instance, P2 stated that “*Now if I had to do a lot of fine motor skill things, like working on a car engine or something like that, it would be a limiting factor*.”

OTs’ primary concerns were related to the stroke-induced physical constraints that may hinder patients from wearing the sensors. Some patients may have severe impairments in fine-hand motor skills in the early stage of the rehabilitation process. In this case, instrumenting the ring sensor on the unaffected finger would require a certain level of dexterity on the affected arm. T11 explained, “*Stroke survivors have to have one [ring sensor] on each hand. They probably aren’t capable of, depending on their limitations, picking it up and putting it on the other hand*.” In addition to lack of motor skills, sensory deficits might make it challenging to don and doff the ring sensors. T11 described “*They could have perceptual problems, so sometimes people don’t recognize a half of their body. Their sensation might be impaired, so they might not be able to feel that it’s on there and not recognize, ‘I need to take this off now.’*” Hence, some stroke survivors with severe fine motor impairments may encounter difficulty instrumenting the finger-worn sensors without the help of caregivers.

Another long-term issue that can affect patients’ adherence to wearing finger-worn sensors is their physiological symptoms. OTs explained that some stroke survivors experience edema (i.e., swelling caused by excessive fluid trapped in body tissues), which results in fluctuation in the patients’ finger size over time. T11 said, “*A lot of people who’ve had cerebral vascular accidents have edema, so they have some swelling in their hands, so being able to fit a ring over the swelling might be a bit of an issue*.” Hence, the ring sensors may not always appropriately fit their fingers. OTs pointed out that stroke survivors should be given options to choose from among different form factors and the body locations they wear the sensors to enhance their adherence to continued and longitudinal use. For instance, T8 stated, “*You should have the capacity for them to use it [for instance] as a wristwatch. There should be other ways in which you can wear a half-glove, and your sensor can go at the back of a half-glove*.”

#### Different preferences in technology

Our analysis suggests that stroke survivors have different but strong preferences for the computing devices to review sensor data. Three stroke survivors (P1, P2, P3) showed positive receptiveness to wearing the ring sensors and self-monitoring the data, but when we asked how they wanted to check the sensor data feedback, they had different opinions. P2 preferred to use his smartphone to review the sensor data. As P2 drives a shuttle for a living, he envisioned reviewing the sensor data frequently during breaks outside. P2 stated that “*I am on the road every day. … I would like to have [the sensor data] available anytime I would like to look at it*.” He did not have difficulty reading materials on his smartphone and routinely used his smartphone for every task as he explained “*So when I have a few free moments, I’m always looking at my phone for emails and text messages and so forth*.” We asked if he would use other devices to review the data using a different device at home (e.g., digital album in his kitchen), he responded that “*[I] probably would see it once or twice a day because [I am] never home*.”

On the other hand, P1 preferred using her laptop because it was easier to see the visual materials—e.g., video streaming as she stated that she watched YouTube videos substantially—and she liked to interact with the physical keyboard. As she continued her explanation, she stressed that she would not use devices other than her laptop. For instance, when it comes to a smartphone, she said “*Well, some things are in other realms, but it’s not a habit I have to do things on the cell phone*.” Similarly, P3 wanted to use her desktop computer when reviewing the sensor data although she was lenient to using her smartphone, “*It’d be nice to have [the sensor data] on my Android cell phone. Actually and mostly on my computer. … My desktop*.” She further explained that she could review the sensor data on her phone, but she emphasized that the data should be presented in a large enough font so that she could see. Given their different preferences in the choice of technology to view their data, the sensor data feedback should ideally be delivered in a cross-platform app, accommodating stroke survivors’ varying degrees of sensory and physical challenges (e.g., vision and fine-hand movements).

#### Comprehensive roles of OTs in using the sensor data

As mentioned earlier, our OT participants showed high interest in engaging with the sensor data. We found two particular roles that OTs were willing to play in interacting with the data: (1) a commentator who identifies meaningful data points and insights from the dataset and explain them to patients and (2) a curator who picks out favorable data segments that could adequately represent patients’ progress in order to maximize benefits from insurance companies.

OTs’ role as a commentator to help stroke survivors understand the data was deemed important because not all performance measures could be relevant for individual patients with unique impairments, and patients may experience difficulty comprehending multi-dimensional information due to their impaired cognitive ability. T14 commented, “*For 90% or 95% [of patients], I would probably just highlight something for them because I think if you give [patients] too much data, they may miss the [therapeutically important] points*.” Similarly, stroke survivors anticipated that OTs would interpret the data and provide better therapy programs. P2 stated, “*If the therapist is receiving that information, and seeing [it], I think they probably would be the ones this information would be most useful to. They would be very good at supplying suggestions of specific exercises to do to enhance better muscular mobilization [based on the sensor data]*.”

Our interview with OTs suggests that they also perceived themselves as a data curator. Each patient’s impairment and recovery patterns are unique. Thus, OTs currently employ the conventional clinical assessment tools that best represent each patient’s therapy-induced improvements in their motor function. OTs explained that, in a similar vein, they would carefully examine various sensor measures, pick the measures that best represent patients’ motor performance and progress, and share it with third-party payers to demonstrate patients’ therapy-induced progress and justify the financial subsidy for patients’ therapy sessions. T2 stated:

*I do not think it [patient’s arm use sensor data] is something that should automatically go to insurances. [I will] use our judgment of ‘Okay, this could be something that could help justify.’ … Same thing with a lot of standardized assessments, like the standardized assessments that I choose to use oftentimes will be the ones that I’m like, ‘Hey, it will show an improvement, and I won’t choose to use this one because it will not show an improvement.’*

## Discussion

### Data as justification

Our study showed that both stroke survivors and OTs envisioned using the sensor data to self-advocate and justify therapy-induced progress in patients’ daily arm performance and receive the financial subsidy from third-party payers for their therapy sessions. Such envisioning is strongly associated with the financial burden of stroke rehabilitation and care in the US. While this finding could be subject to the US-specific context, the cost of stroke-induced care is among the most expensive chronic conditions and a cause of significant financial burden for patients worldwide [65–67]. Such a high-cost burden serves as a major barrier that prohibits stroke survivors from receiving longitudinal rehabilitation therapies. OTs perceived that conventional assessment tools are inappropriate to frequently and objectively track changes in the arm performance level and therapeutic outcomes [68, 69]. Our findings suggest that the sensor data could support these unmet needs. At the same time, further research seems inevitable to establish more clinical references so that patients, OTs, and third-party payers can implement evidence-based rehabilitation [70], which the interviewed OTs also emphasized in our study. One promising direction of future research would be to establish normative data as well as minimum clinically important differences (MCID) for sensor-based measures, similarly to conventional assessment tools such as the Fugl-Meyer Assessment [71, 72]. Therapists could use the normative data and MCID to gauge patients’ relative ability (i.e., severe, moderate, mild, normal) with respect to that of a healthy population and determine if observed changes in assessment scores represent therapeutically meaningful improvements, respectively. The establishment of these data could help improve the credibility of sensor measures on patients’ performance improvements, which in turn will accelerate the translation of sensor-based performance measures into routine rehabilitation therapy.

Despite the anticipated therapeutic benefits of sensor-based performance measures, a chance of intentional data misuse cannot be ruled out. For instance, stroke survivors and rehabilitation facilities (e.g., nursing homes) may purposefully misuse the measures to claim financial subsidies from third-party payers. As reported in prior studies, sensor users may cheat by exaggerating movements on their affected arm by shaking the sensor periodically [73], and nursing homes may make false medical claims to increase their revenue [74]. One solution would be to develop an intelligent algorithm to detect such undesirable use scenarios [75], which will help third-party payers to trust and adopt the patient-generated performance measures.

### Misrepresenting daily performance

In the real-world context where the finger-worn sensor system is deployed, stroke survivors—potentially with the help of caregivers—are expected to properly operate the sensors and collect data without close supervision of clinicians. In uncontrolled settings, there exists a chance that sensors are inappropriately operated, resulting in the misrepresented patients’ daily performance. For instance, as many stroke survivors experience cognitive (e.g., memory and attention) [76] and sensory disorders (e.g., visual and tactile) [77], stroke survivors may accidentally don the sensors on the opposite sides (e.g., the left ring on the right finger and the right ring on the left finger). This may lead to an incorrect representation of patients’ arm performance. One solution would be to design the appearance of sensors in a way that stroke survivors can easily discern the sides (e.g., coloring or shaping the left and right sensors distinctively). Another approach would be to implement anomaly detection algorithms to detect if the measured sensor data show notably different patterns compared to the past [78]. Furthermore, the detected patterns could provide opportunities for OTs to examine reasons for anomalies observed in the data and share clinical interpretations with colleague therapists or other clinicians (e.g., MD) [34]. Providing local archives, notes, and data export can be useful for OTs to manage such data patterns and cases.

### Data analytics and feedback tools for OTs and patients

Our findings revealed the necessity of more sophisticated data analytics tools for OTs and visualization feedback for stroke survivors. All the OTs we interviewed perceived themselves as a data curator and expressed intention to navigate a set of performance measures of different duration (e.g., a day, week, month or year) and scale (e.g., minutes, hours, days), identify therapeutically meaningful measures and subsets of data, and share them with their patients and third-party payers. Furthermore, they envisioned using the identified data points to customize patient visits and therapy programs. However, without proper tools that assist OTs in investigating the data, it will be time-consuming to navigate a large volume of data and identify therapeutically meaningful measures and data points. Prior studies reported that clinicians felt overwhelmed when patient-generated data of a sheer size were presented, which would make it difficult for clinicians to extract relevant information and even discourage them from adopting the sensor system at all [45, 79]. Hence, the next logical step is to devise a data analytics tool that goes beyond a mere visualization tool [45, 80] that can automatically analyze the sensor data. Then, the system could present a list of measures and data segments that may convey therapeutically important information, from which OTs can utilize to personalize therapy programs or to share with individual patients.

Similar to a recent study on experiential information [59], our OT participants stated the importance of knowing stroke survivors’ contextual information when interpreting their sensor data. Specifically, the arm use data do not provide activity semantics—for example, what they were doing during peaks and troughs—which may be important cues in devising a personalized therapy program. To obtain rich and accurate contextual information, stroke survivors may be involved in providing activity labels (i.e., annotating their sensor data) [45, 46, 80, 81]. As many stroke survivors are older adults and have some degree of cognitive and motor impairments, an application can send individually-tailored, data-driven notifications to remind patients of logging contextual information [82, 83], to which stroke survivors can respond via multimodal interactions (e.g., touchscreen or speech-based data capture).

Stroke survivors are the other major users of the sensor data. Our findings suggest that easy-to-understand, adaptable feedback needs to be provided to stroke survivors. In addition to the summary of arm use performance, the feedback could also convey personalized therapy goals, programs (i.e., strategies to incorporate the affected arm in daily living), and specific data points that OTs deemed therapeutically meaningful for patients to review. Furthermore, various features to support self-management can be integrated into the patient-facing system. For instance, positive feedback can compliment patients when they meet the set therapy goals, and reminders can alert patients to use their affected arm more when they under-use it. However, prior studies stop short at investigating simple visualization tools or reminders [35, 36, 52, 53] and do not take account of comprehensive information that is revealed in this study. While implementing tools with the above-mentioned functionality, the unique characteristics of stroke survivors should be considered (e.g., age and impairments). A recent study by Wu et al. suggests that a new set of design guidelines for visualization should be devised for a population with developmental disabilities [84]. Similarly, substantial research efforts seem necessary to implement a feedback system that can present comprehensive information to stroke survivors with cognitive and motor impairments in a manner that can be easily browsed and understood.

### Promoting the use of sensor for self-monitoring

Our stroke participants’ willingness to wear the ring sensors was largely conditioned on external motivation (e.g., therapists’ endorsement and justifying the financial subsidy of third-party payers). In other words, it is not clear if stroke survivors will be willing to continue to wear the sensors to self-monitor their own performance in the absence of external motivation (e.g., when the financial subsidy from third-party payers is depleted and their subsidized therapies are discontinued). According to Nieboer et al., nearly 40% of stroke survivors in their study did not adhere to the use of the gait monitoring sensor system at all although they acknowledged the therapeutic benefits of self-monitoring [85]. Similarly, stroke survivors may discontinue the voluntary use of the ring sensors, which may hinder the improvement and maintenance of the therapy-induced motor performance.

Given the importance of continuous engagement with self-monitoring, we may need to incorporate nudging strategies to motivate stroke survivors to continue self-management (e.g., ambient feedback, just-in-time prompts, and social comparison) [86]. For instance, a prior study with older adults demonstrated that an ambient self-monitoring display in the home setting could reinforce older adults’ adherence to longitudinal medication for ten months [87]. In our context, stroke survivors’ performance measures can be displayed in various mediums in the home setting (e.g., digital frame) so that their exposure to arm activity information could reinforce their adherence to self-monitoring. Another approach would be to use social comparisons. In our study, P2 stated that he would want to share his performance data with “*some of [his] stroke survivor friends that [he] go[es] to a stroke support group with*” as they often share “*their lifestyle, … what [they] do*.” Hence, a smartphone app can support stroke survivors to share their performance data with whom they choose, which may help stroke survivors engage in self-monitoring.

## Limitations and future work

Our study has several limitations worth discussing. First, the positive receptiveness of stroke survivors towards using the proposed ring sensors could be subject to the participated stroke survivors. Three out of four patients received some level of higher education and used computing devices daily. Considering that only about 50% of older adults receive higher education [88] and only 38%–44% of older adults use computing devices (e.g., laptop and smartphones) on a daily basis [89], stroke survivors with less educational level and experience of using digital devices may show responses that are different from what is reported in this study. Second, the stroke survivors’ responses are based on their experience for a short duration (i.e., two days of daily living). A longer duration use may surface new positive or negative experiences with the sensors that were not reported in this study. Hence, a future study should consider a longer deployment that matches the duration of the actual rehabilitation service. Third, while stroke survivors in our study expressed their willingness to wear the sensors, they asked for a smaller form factor. For example, the embedded system design could further optimize the form factor (e.g., embedding a battery that is shaped like a ring). Fourth, OTs’ responses are based on the two days of data from a single stroke survivor. As we deployed the sensors to stroke survivors for two days, a subset of the study probes, especially for the week, month, and year data (Fig 3(b)–3(h)), were fabricated. While the findings in this study provide valuable insights into how the patient-generated data could be used in routine practice, a real-world study involving a larger number of patients with various types and severity of impairments can further reveal more diverse strategies of OTs that we may not have found in this study. Hence, it warrants a future study to deploy the fully-functioning, interactive data visualization system in the actual rehabilitation setting for a prolonged period and investigate how patients’ and OTs’ perspectives on the usefulness of the patient-generated data evolve over time.

## Conclusion

In this work, we reported how stroke survivors and OTs envisioned the use of in-situ arm movement data in achieving patient-centered rehabilitation therapies in the outpatient setting. To situate our participants, we leveraged a pair of finger-worn accelerometers to collect stroke survivors’ actual data, which we used to create design probes for stroke survivors and OTs, respectively. Based on the semi-structured interviews we facilitated with the design probes, we unveiled a detailed account of (1) the receptiveness of stroke survivors and OTs in the light of overcoming their practical challenges in the conventional outpatient rehabilitation setting, (2) OTs’ strategies to utilize patient-generated sensor data to provide patients with personalized therapy programs of greater quality, and (3) practical challenges to be considered to accelerate the integration of wearable system into their practice. These findings offer promising directions for the design of wearable solutions that can assist the essential goal of helping stroke survivors—and more broadly, individuals suffering from hemiparesis—achieve independent living.

## Supporting information

S1 File Author details and COREQ checklist. (DOCX)

S2 File Stroke survivor interview script. (DOCX)

S3 File Probe for therapist interview. (PDF)
