# Supplementary material for: Envisioning the use of in-situ arm movement data in stroke rehabilitation: Stroke survivors’ and occupational therapists’ perspectives
Source: PLoS One. 2022 Oct 20;17(10):e0274142. doi: 10.1371/journal.pone.0274142 (PMC9584451; doi:10.1371/journal.pone.0274142)
Supplement: S1 File — (DOCX) [file pone.0274142.s001.docx]

**Supporting information file S1: Author details and COREQ checklist**

1. **Author details**

Hee-Tae Jung (HJ), lead author, PhD, male was a postdoctoral research fellow at the University of Massachusetts Amherst when the study was conducted. HJ has an educational background in Information and Computer Sciences and was trained as part of his PhD in qualitative methods. HJ has experience in investigating the needs of stakeholders and developing technological solutions to enhance the quality of rehabilitation services for people with chronic conditions.

Yoojung Kim (YK), female, was a PhD candidate at Seoul National University when the study was conducted. YK has a background in Human-Computer Interaction and was trained as part of her PhD in qualitative methods. YK has experience in investigating the needs of stakeholders and developing technological solutions to support medical services and clinician-patient interaction.

Juhyeon Lee (JL), female, was a PhD student at the University of Massachusetts Amherst when the study was conducted. JL has an educational background in Electrical Engineering and Computer Science. JL has experience in developing remote monitoring systems for people with chronic conditions.

Sunghoon Ivan Lee (SIL), PhD, male, is an assistant professor at the University of Massachusetts Amherst. SIL has a background in Electrical Engineering, Computer Science, and Rehabilitation Science. SIL has experience in developing remote monitoring systems for people with chronic conditions, such as stroke, arthritis, and ataxia.

Eun Kyoung Choe (EKC), PhD, female, is an associate professor at the University of Maryland, College Park. EKC has a background in Human-Computer Interaction and Health Informatics. EKC has experience in developing technological solutions to support self-tracking in diverse healthcare contexts, such as sleep, diet, and exercise.

1. **COREQ (COnsolidated criteria for REporting Qualitative research) Checklist**

| **Topic** | **Item No.** | **Guide Questions/Description** | **Reported on Page No.** |
| --- | --- | --- | --- |
| **Domain 1: Research team and reflexivity** |  |  |  |
| *Personal characteristics* | | |  |
| Interviewer/facilitator | 1 | Which author/s conducted the interview or focus group? | 10 A subset of the authors conducted the interview. No more than three authors participated at a time to maintain amiable atmosphere. |
| Credentials | 2 | What were the researcher’s credentials? E.g. PhD, MD | S1 HJ, YK, SIL, EKC hold PhD. JL is a PhD student. |
| Occupation | 3 | What was their occupation at the time of the study? | S1 HJ was a postdoctoral research fellow, YK was a PhD candidate, JL was a PhD student, SIL and EKC were professors. |
| Gender | 4 | Was the researcher male or female? | S1 HJ, SIL were male. YK, JL, EKC were female. |
| Experience and training | 5 | What experience or training did the researcher have? | S1 HJ, JL, SIL had experience in quantitative and qualitative research around the topics of biomedical and health informatics. YK and EKC had experience in quantitative and qualitative research around the topics of health-related human-computer interaction. |
| *Relationship with participants* |  |  |  |
| Relationship established | 6 | Was a relationship established prior to study commencement? | 6 No prior relationship. |
| Participant knowledge of the interviewer | 7 | What did the participants know about the researcher? e.g. personal goals, reasons for doing the research | 8-10 Research motivation and goals were explained to the study participants in the beginning. |
| Interviewer characteristics | 8 | What characteristics were reported about the interviewer/facilitator? e.g. Bias, assumptions, reasons and interests in the research topic | S1 Researchers have been involved in developing health technologies and investigating the experience by target stake holders (e.g., practitioners and patients). |
| **Domain 2: Study design** | | |  |
| *Theoretical framework* |  |  |  |
| Methodological orientation and Theory | 9 | What methodological orientation was stated to underpin the study? e.g.  grounded theory, discourse analysis, ethnography, phenomenology, content analysis | 10-11 Thematic analysis was conducted. |
| *Participant selection* |  |  |  |
| Sampling | 10 | How were participants selected? e.g. purposive, convenience, consecutive, snowball | 7 All the participants were selected in a first-come-first-served manner based on their voluntary contacts. |
| Method of approach | 11 | How were participants approached? e.g. face-to-face, telephone, mail, email | 8-10 Recruiting materials were posted and distributed online. |
| Sample size | 12 | How many participants were in the study? | 5-7 Four stroke survivors and 15 occupational therapists participated in the study. |
| Non-participation | 13 | How many people refused to participate or dropped out? Reasons? | 5 No participants were dropped out or refused. |
| *Setting* |  |  |  |
| Setting of data collection | 14 | Where was the data collected? e.g. home, clinic, workplace | 8-10 With stroke survivors, the data was collected at their preferred location (e.g., their home). With occupational therapists, the data was collected on zoom. |
| Presence of nonparticipants | 15 | Was anyone else present besides the participants and researchers? | 8-10 No one was present other than the participants and researchers. |
| Description of sample | 16 | What are the important characteristics of the sample? e.g. demographic data, date | 6-7 Detailed information was provided in **Table 1 and 2** in the **manuscript**. |
| *Data collection* |  |  |  |
| Interview guide | 17 | Were questions, prompts, guides provided by the authors? Was it pilot tested? | 8-10 Semi-structured interview questions were prepared and asked by the researchers. The tutorial and interview questions were pilot tested by a healthy young adult and two occupational therapists. |
| Repeat interviews | 18 | Were repeat interviews carried out? If yes, how many? | No The interview session was once for each participant. |
| Audio/visual recording | 19 | Did the research use audio or visual recording to collect the data? | 10 All the interviews were audio-recorded. |
| Field notes | 20 | Were field notes made during and/or after the interview or focus group? | No No field notes were made. |
| Duration | 21 | What was the duration of the interviews or focus group? | 8-9 The interview with stroke survivors lasted for about an hour. The interview with occupational therapists lasted for about an hour and a half. |
| Data saturation | 22 | Was data saturation discussed? | 10 The interview with occupational therapists was terminated when all the authors agreed that the data reached saturation. |
| Transcripts returned | 23 | Were transcripts returned to participants for comment and/or correction? | No The transcripts were not returned to the study participants. |
| **Domain 3: analysis and findings** | | |  |
| *Data analysis* |  |  |  |
| Number of data coders | 24 | How many data coders coded the data? | 10-11 All the authors participated in the data coding. |
| Description of the coding tree | 25 | Did authors provide a description of the coding tree? | S2 The coding book is provided as Supplement file (S2). |
| Derivation of themes | 26 | Were themes identified in advance or derived from the data? | 11 Themes were derived from the data. |
| Software | 27 | What software, if applicable, was used to manage the data? | 10 ATLAS.ti cloud was used when analyzing the data. |
| Participant checking | 28 | Did participants provide feedback on the findings? | No  The findings were not provided to the study participants. |
| *Reporting* |  |  |  |
| Quotations presented | 29 | Were participant quotations presented to illustrate the themes/findings?  Was each quotation identified? e.g. participant number | 11-19 The quotations that support the derived themes were presented in the Findings section. |
| Data and findings consistent | 30 | Was there consistency between the data presented and the findings? | 11-19 The findings reported in the manuscript are consistent with the themes derived from the data. |
| Clarity of major themes | 31 | Were major themes clearly presented in the findings? | 11-19 Each theme was presented with a descriptive title and the summary of the theme was articulated in the beginning of each subsection. |
| Clarity of minor themes | 32 | Is there a description of diverse cases or discussion of minor themes? | 11-19 One stroke survivor had symptoms that are different from the rest of the participating stroke survivors, which was reported in the Findings. |

Developed from: Tong A, Sainsbury P, Craig J. Consolidated criteria for reporting qualitative research (COREQ): a 32-item checklist for interviews and focus groups. *International Journal for Quality in Health Care*. 2007. Volume 19, Number 6: pp. 349 – 357
