# Supplementary material for: Envisioning the use of in-situ arm movement data in stroke rehabilitation: Stroke survivors’ and occupational therapists’ perspectives
Source: PLoS One. 2022 Oct 20;17(10):e0274142. doi: 10.1371/journal.pone.0274142 (PMC9584451; doi:10.1371/journal.pone.0274142)
Supplement: S2 File — (DOCX) [file pone.0274142.s002.docx]

# A. Ice-breaking & Onboarding

1. Short introduction
   1. Research team & me: Hello, I am [name] and a part of a multi-institutional research team. [universities and a company] are involved in this study.
   2. Research goal: The goal of this research is to develop a wearable-sensor that can monitor how much you use your affected and unaffected arms in your daily living and visually provide you with the information.
2. Disease history
   1. When was your last stroke?

B. Living pattern & Technology proficiency

1. When you are home, where do you spend most of your time? (e.g., bed room, living room, etc.)
2. On a given day, how long do you stay outside your home?
   1. Where and how do you go?
3. Technology Access and Use
   1. Environment:
      1. Do you have any access to the Internet?
         1. Yes/No?
         2. Wifi/Data plan?
      2. Personal Computer
         1. Do you have one? Yes/No
         2. Do you use it? Yes/No
      3. Conventional cellular phone
         1. Do you have one? Yes/No
         2. Do you use it? Yes/No
      4. Smartphone
         1. Do you have one? Yes/No
         2. Do you use it? Yes/No
      5. Tablet
         1. Do you have one? Yes/No
         2. Do you use it? Yes/No
   2. Proficiency
      1. Computer (Potential medium exploration)
         1. Can you use a computer keyboard to type?
            1. Yes/No/Haven’t tried
         2. Can you use a mouse?
            1. Yes/No/Haven’t tried
      2. Mobile (Basic proficiency)
         1. Can you make a phone call?
            1. Yes/No/Haven’t tried
         2. Can you text someone?
            1. Yes/No/Haven’t tried
      3. Computer and mobile (Potential medium exploration)
         1. Can you web-browse?
            1. Yes/No/Haven’t tried
            2. Using which device?
         2. Can you use a touchscreen interface (e.g., smartphone or tablet)?
            1. Yes/No/Haven’t tried
         3. Can you send emails?
            1. Yes/No/Haven’t tried
            2. Using which device?
         4. Can you watch videos?
            1. Yes/No/Haven’t tried
            2. Using which device?
4. Do you monitor and keep track of the use amount of both your arms in ADL?
   1. If you do,
      1. How do you do it now?
      2. Do you use any commercial tracking products, such as Fitbit?
      3. Do you receive any help from your therapists or caregivers (e.g., your spouses) when tracking?
   2. If you don’t,
      1. Why do you not monitor or keep track of it (e.g., no desire, habit)?
      2. Do you want to do it?
      3. What do you want to be monitored and tracked more specifically?
5. Do you do rehabilitation exercises at home?
   1. If you do,
      1. What exercises do you do?
      2. How much exercise do you do (e.g., 30 minutes, 1 hour)?
      3. When do you do those exercises (e.g., in the morning, afternoon, evening)?
      4. Do you monitor and track the exercises and the time duration you do them?
      5. Do you track the use amount of both your arms while performing rehabilitation exercises?
      6. Do you receive any help from therapists or caregivers (e.g., your spouses) when tracking?
   2. If you don’t, **No**
      1. Why do you not exercise (e.g., no desire, habit)?
      2. Do you want to do exercises?
      3. Do you want to monitor and track it (e.g., the type or the duration of exercises, the use amount of both arms)?
      4. What do you want to be monitored and tracked more specifically?

#

# Data-driven feedback visualization:

1. **Insight evaluation**
   1. Which of the following actions do you believe would help achieve your daily goal (choose all that apply)?
      1. Use the affected limb more than the unaffected limb
      2. Use the unaffected limb more than the affected limb
      3. Use both limbs equally
   2. **Interesting points:** Do you find the visualized reports useful? What did you like about the visualized reports you saw?
   3. **Preference**
      1. (Showing the three pairs of six reports) Please rank the three reports based on your preference. Can you tell us the reasons?
      2. (for each visualization) Could you tell me what aspects you like in this visualization (sample) if any? Why?
      3. (for each visualization) Could you tell me what aspects you don't like in this visualization (sample) if any? Why?
   4. **Suggestions:** Do you have any suggestions to improve these more interesting/useful/helpful/ for you?
2. **Preferred modality of data-driven feedback**
   1. From which device do you want to receive this kind of feedback? (e.g., mobile phone, tablet PC, laptop (e.g., website/email), TV, paper via mail, IoT devices (e.g., always-on device on a table)
      1. Why?
      2. When and where would you like to receive visual feedback?
3. **A trade-off between perceived benefits and the burden of tracking (acceptability)**
   1. How willing are you to wear sensors to get these types of feedback? Why? How long?

**C. Open-ended questions**

1. If there were no restrictions or limitations, what other data about yourself-- (status, performance in activities) do you want to know?
   1. For which activity (e.g., ADLs or therapeutic exercises)?
   2. For which purpose?
2. Who else do you think can access this data? (e.g., caregiver, therapist) - [ Privacy vs. Utility trade-off ]
   1. Would you have any privacy concerns?
   2. Why? Why not?
   3. (Optional) How much / how detailed information would you be willing to share with [caregiver|therapist]?
3. The current ring sensor you wore is a prototype. Based on your experience of wearing it, how can it be improved? (e.g., Design? Size? Thickness? Weight? Comfort?)
4. Would you feel comfortable meeting other people while wearing sensors?

# **D. Closing**

1. Do you have any suggestions/feedback in general?
