# Supplementary material for: Envisioning the use of in-situ arm movement data in stroke rehabilitation: Stroke survivors’ and occupational therapists’ perspectives
Source: PLoS One. 2022 Oct 20;17(10):e0274142. doi: 10.1371/journal.pone.0274142 (PMC9584451; doi:10.1371/journal.pone.0274142)
Supplement: S3 File — (PDF) [file pone.0274142.s003.pdf]

Supporting Therapists' Practice

Using Sensor-Based Patient Arm Movement Information

University of Massachusetts
University of Maryland
Harvard Medical School

What Are Current Challenges?

Capacity

Learned and practiced in Rehabilitation Setting

≠

Performance

Transfer of learned skills in Daily Living

Research Goal

What Are Current Challenges?

How

Patients

Use Their Arms

Outside Hospitals?

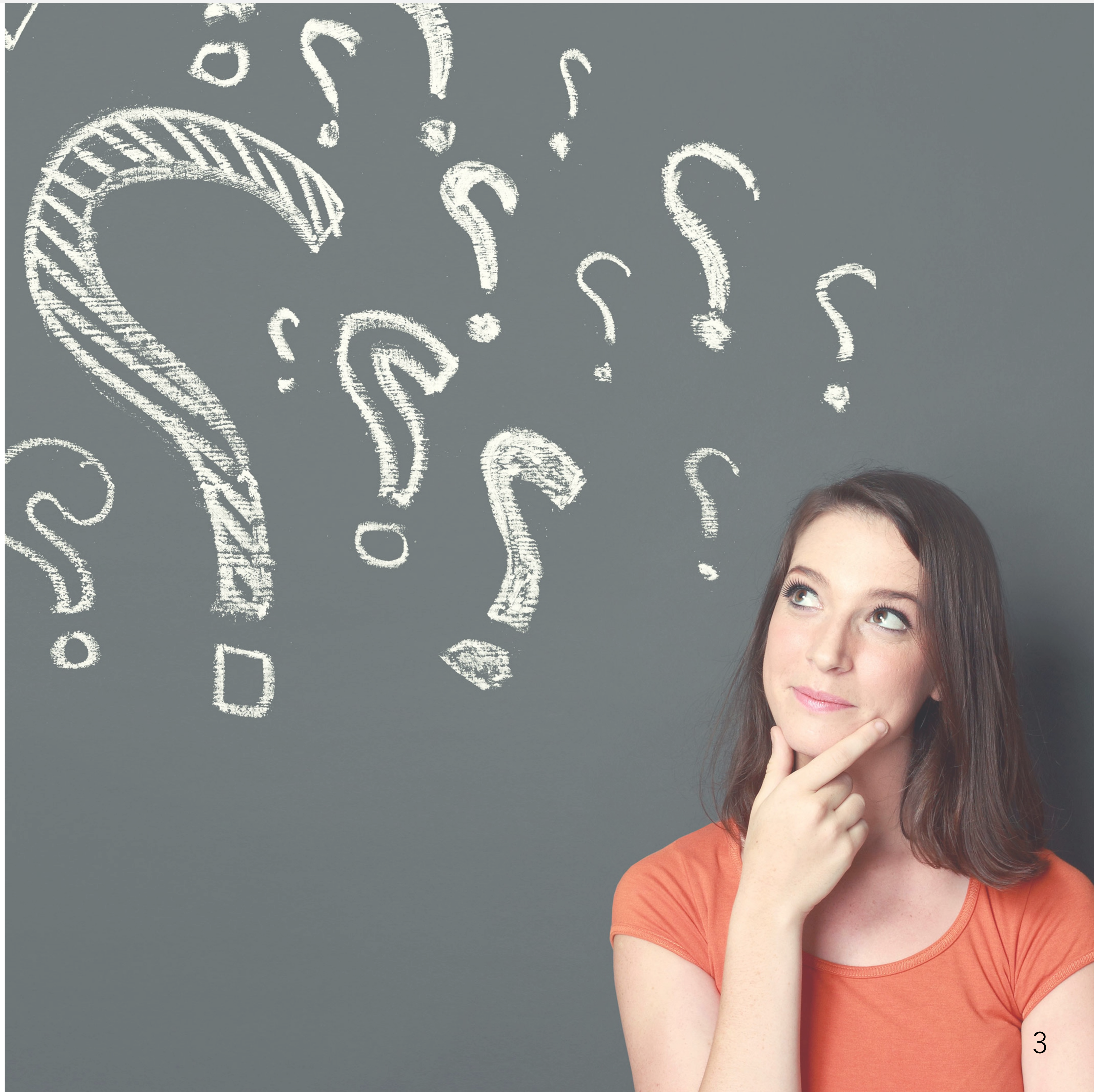

What Does Our Research Aim?

Encourage **Patients** to use their affected arm during activities of daily living

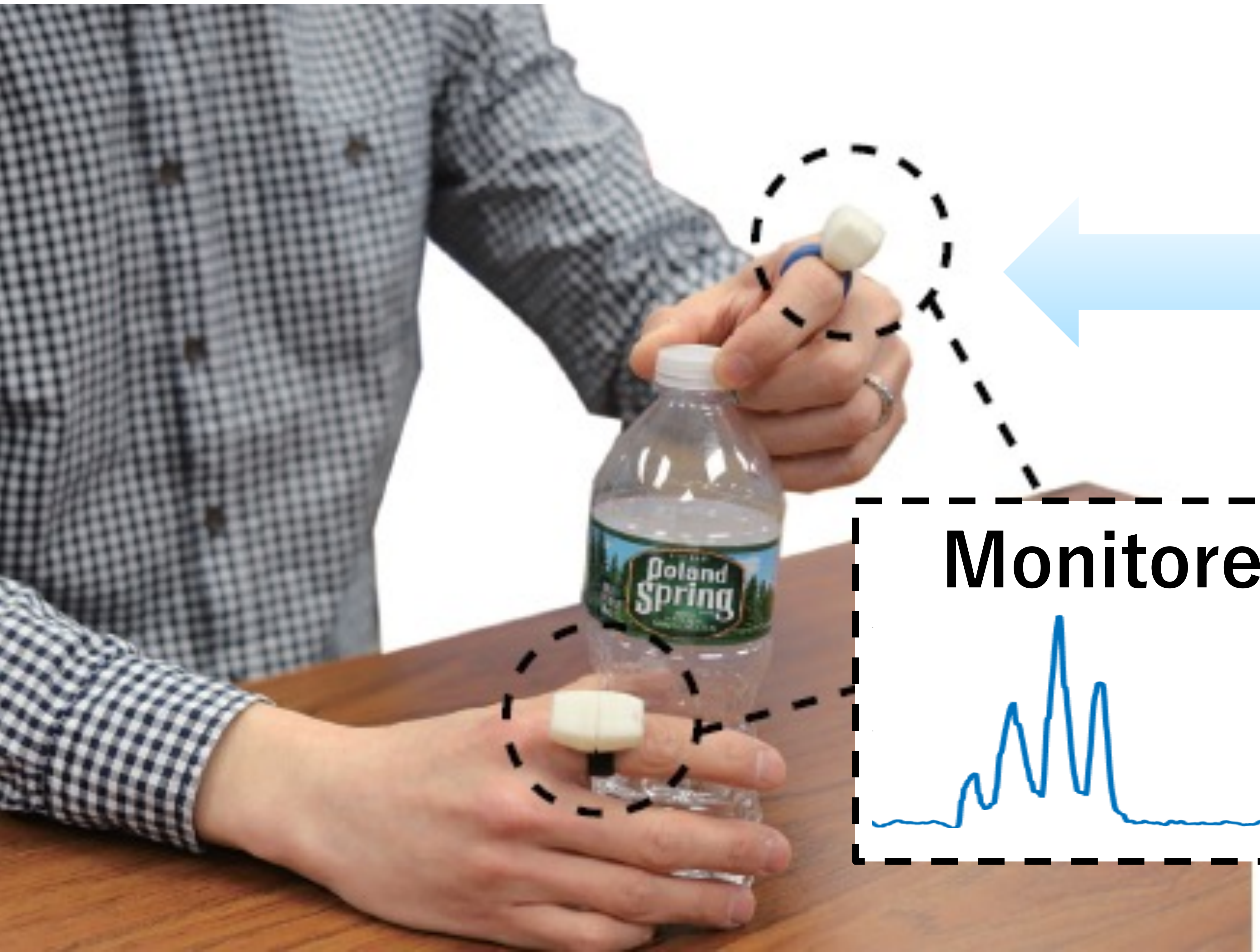

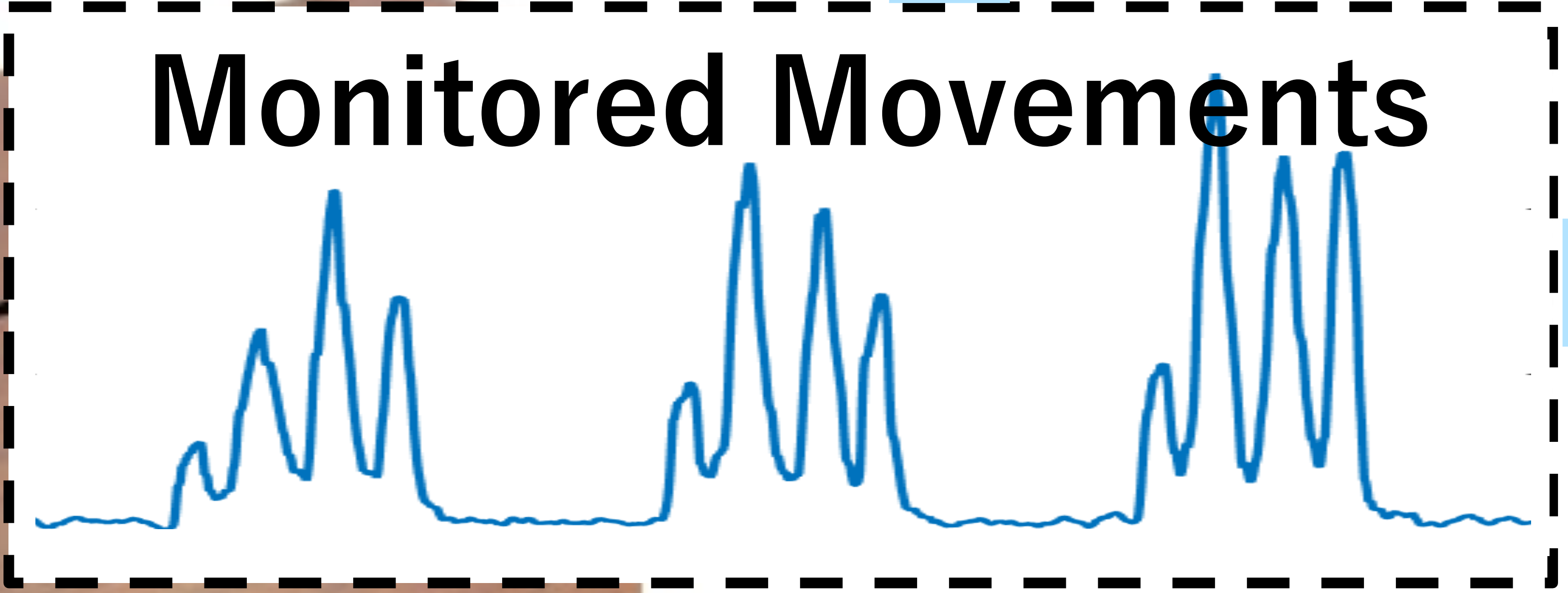

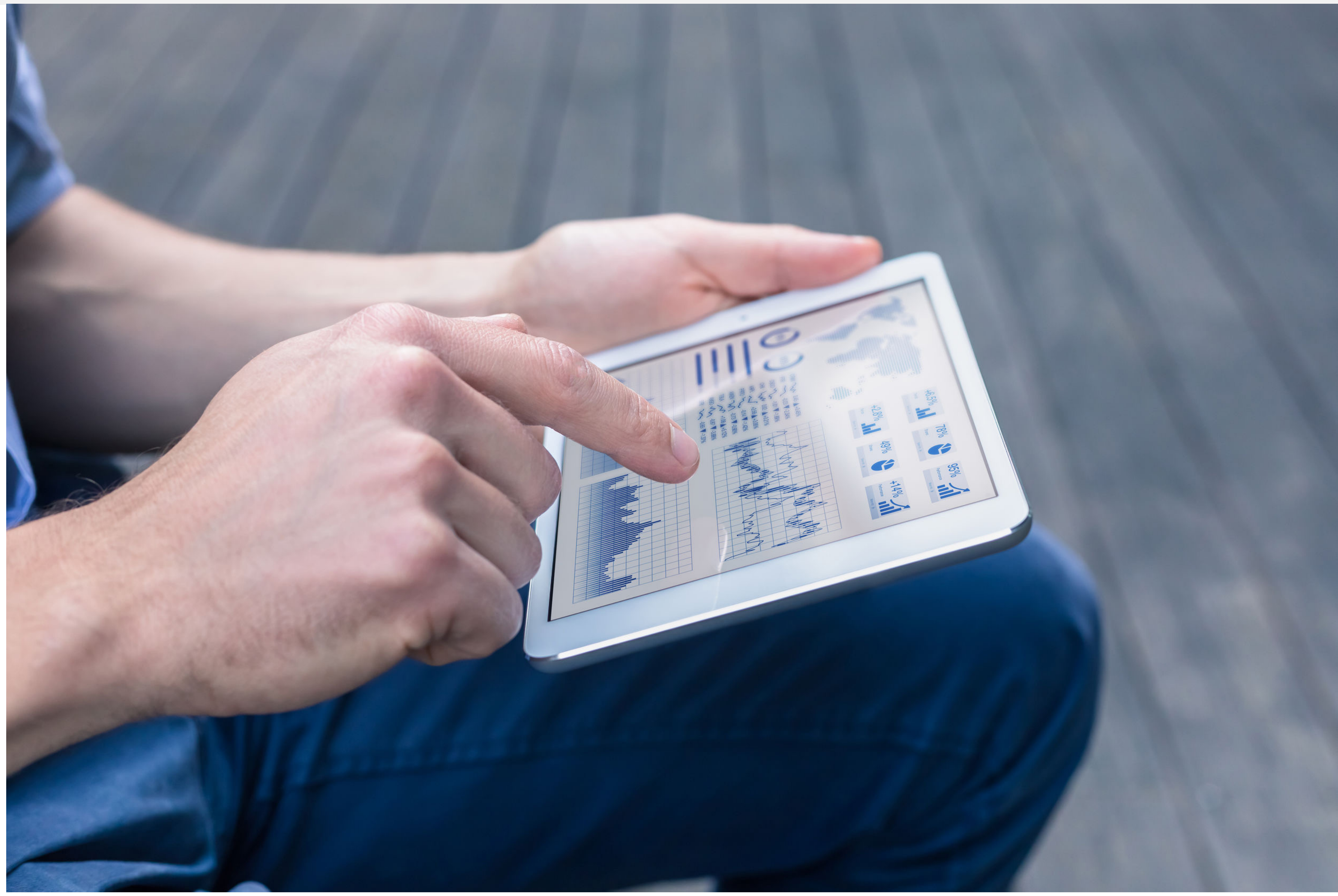

Help **Therapists** Monitor Patients and Personalize Therapy

Ring Sensors

What Does Our Research Aim?

Encourage **Patients** to use
arm during activities of dail

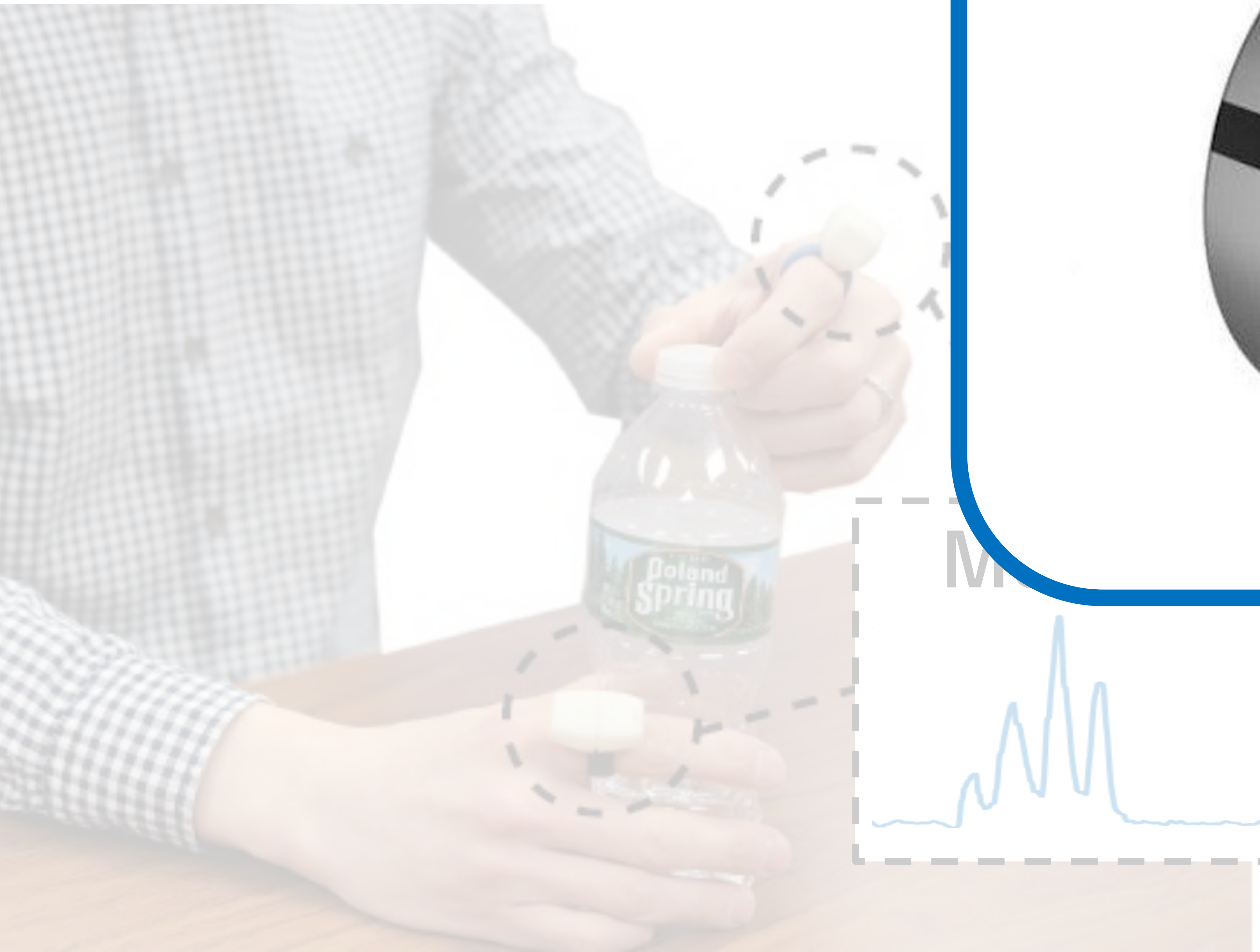

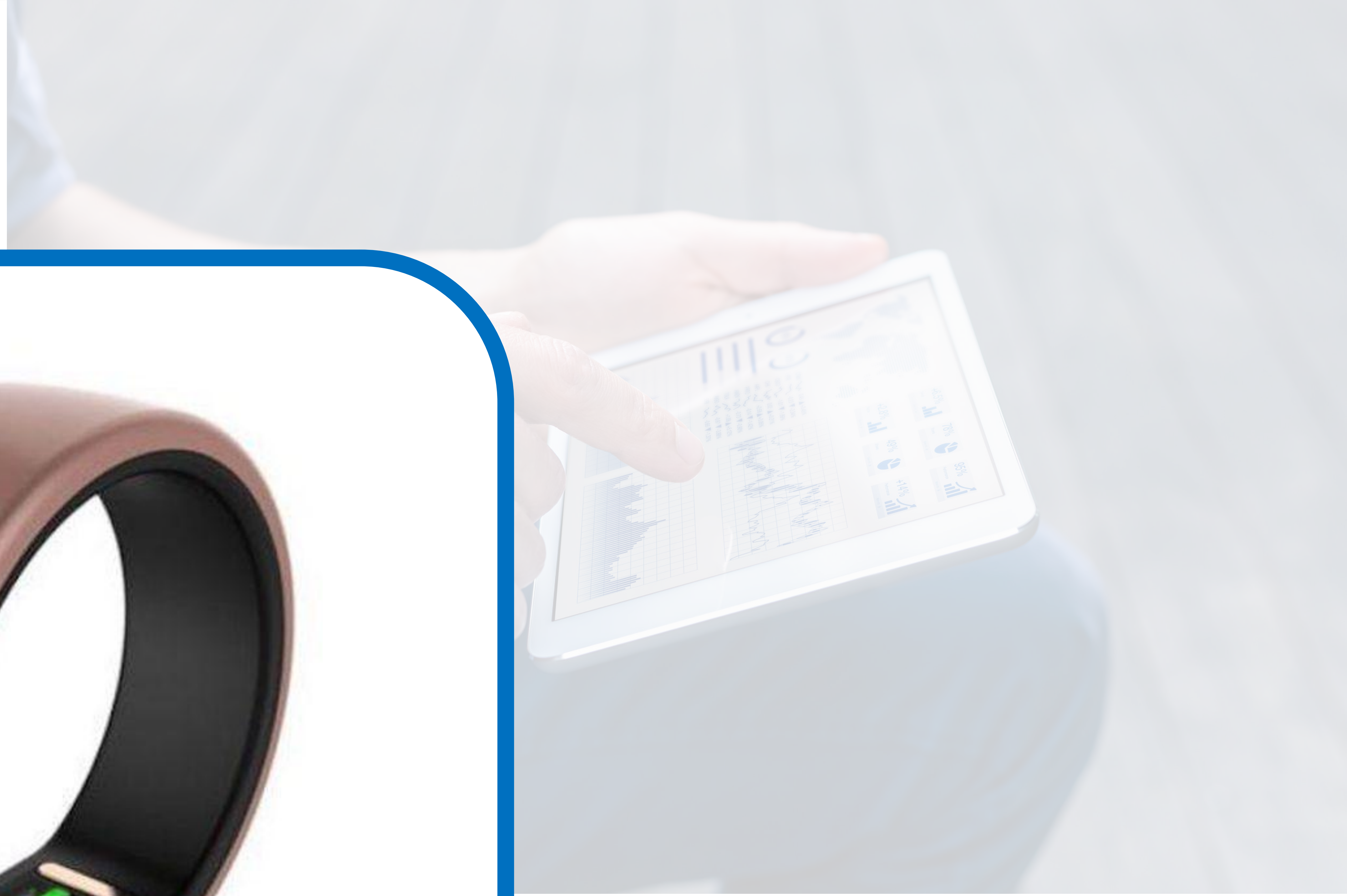

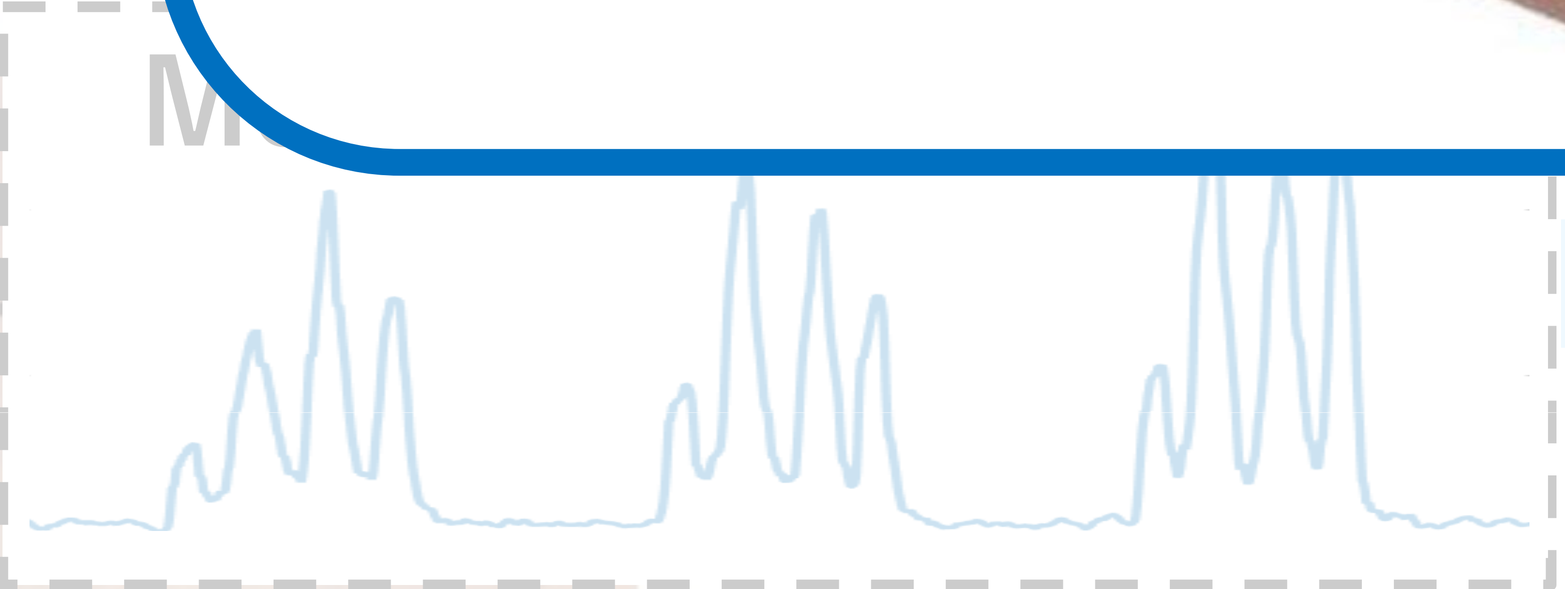

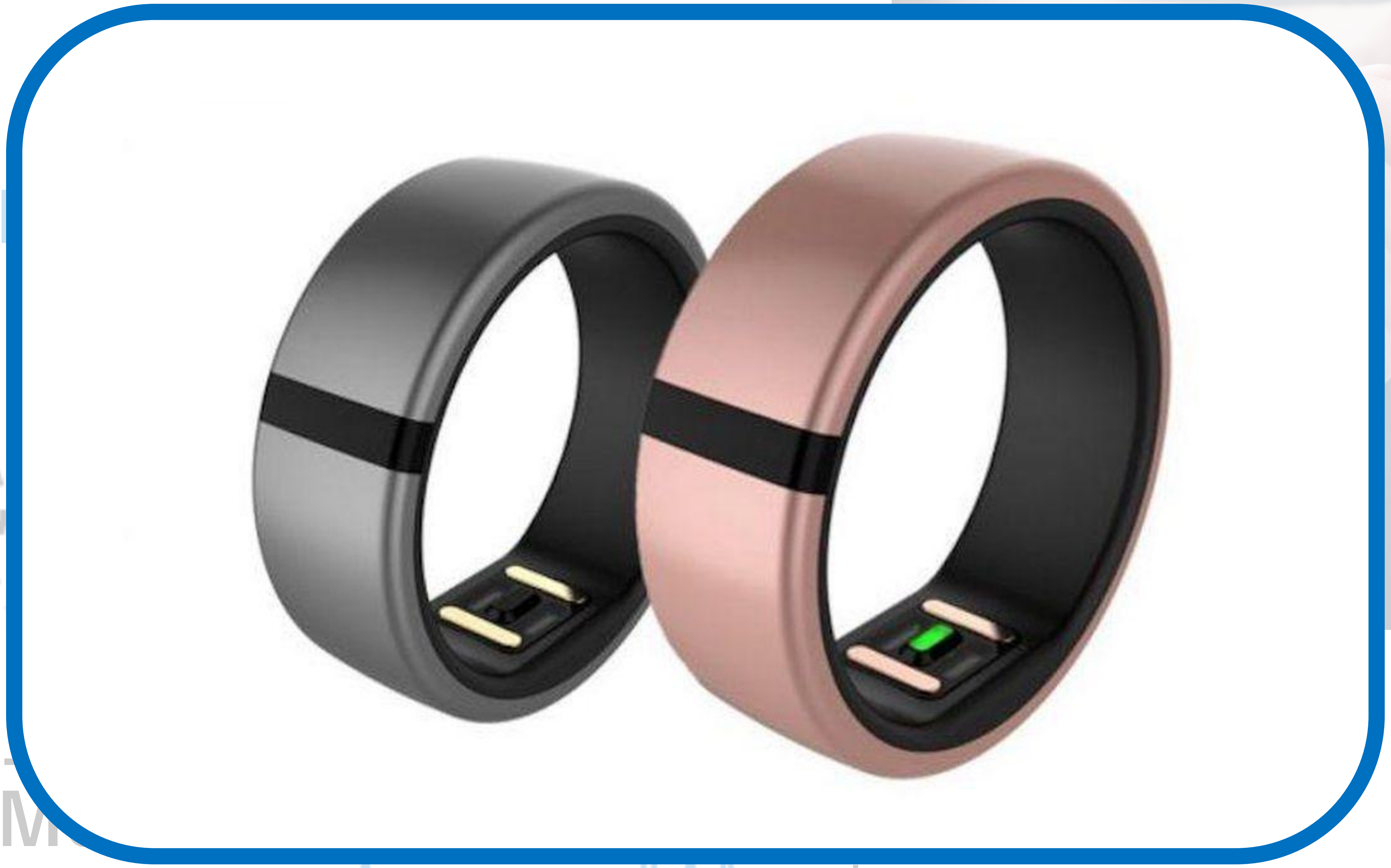

Help **Therapists**
Monitor Patients
and Personalize
Therapy

Research Goal

What Is This Study For?

- Typical practices of outpatient therapy session
- How you make use of the sensor-generated patient movement information
- How such usage affects therapy practices and their quality

Warm Up

What Is Your Typical Therapy Practice When Seeing Outpatients?

A Number of therapy sessions or patients

B Assessment of patients’ function in therapy sessions (standardized tools)

C Remote follow-ups between therapy sessions

Warm Up

What Is Your Typical Therapy Practice When Seeing Outpatients?

Rehabilitation Therapy Routine

- **A** Assess patients' motor status/recovery
- **B** Evaluate adherence
- **C** Identify attainable therapeutic goal
- **D** Administer in-person rehabilitation therapy
- **E** Prescribe home-exercise

What Information Can We Provide Using Sensor Data?

- **Ratio** — Ratio of Affected Limb Use
- **A-Duration** — Duration of Affected Limb Use
- **B-Duration** — Duration of Bilateral Limb Use

Patient Profile

Meet James, Our Stroke Patient

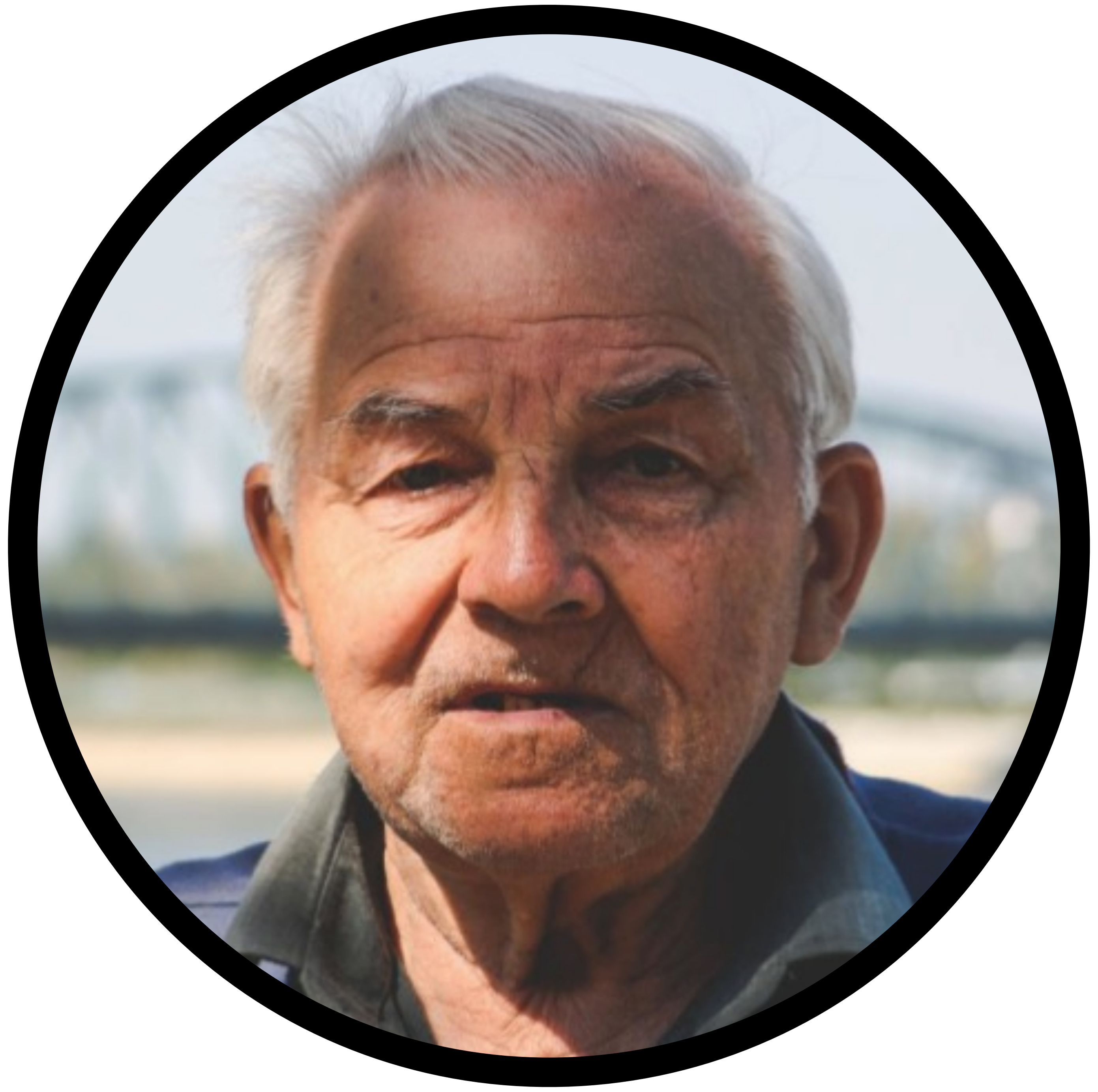

James (74)

**FMA Total (Capacity)**	0 — severe — moderate — mild — 66; marker at 41
**FMA Distal | Proximal**	**19** / 42 points | **22** / 24 points
**MAL Quantity (Performance)**	0 — 5; marker at 0.65
**MAL Quality (Performance)**	0 — 5; marker at 0.68
**Dominant & Affected Side**	• Right-handed<br>• Right hemiparesis for **11 years**
**Living Patterns**	• Lives with his wife<br>• Ambulates with an ankle-foot orthosis<br>• Driving a shuttle for living
**Hobby**	• Web-surfing

Ratio of Affected Limb Use

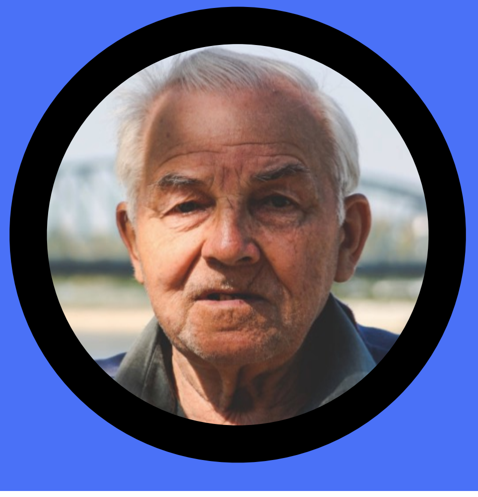

Ratio — Ratio of Affected Limb Use

A-Duration — Duration of Affected Limb Use

B-Duration — Duration of Bilateral Limb Use

Ratio

Average Ratio for 2 Days

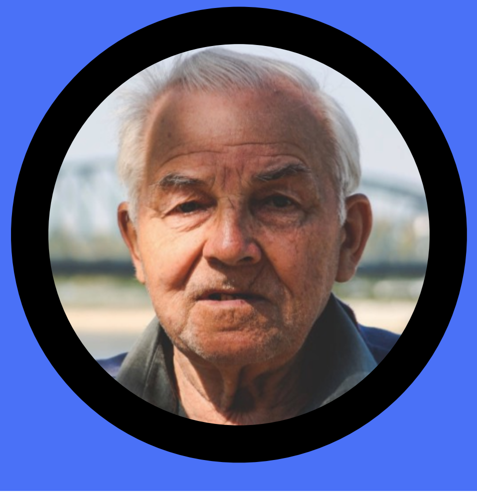

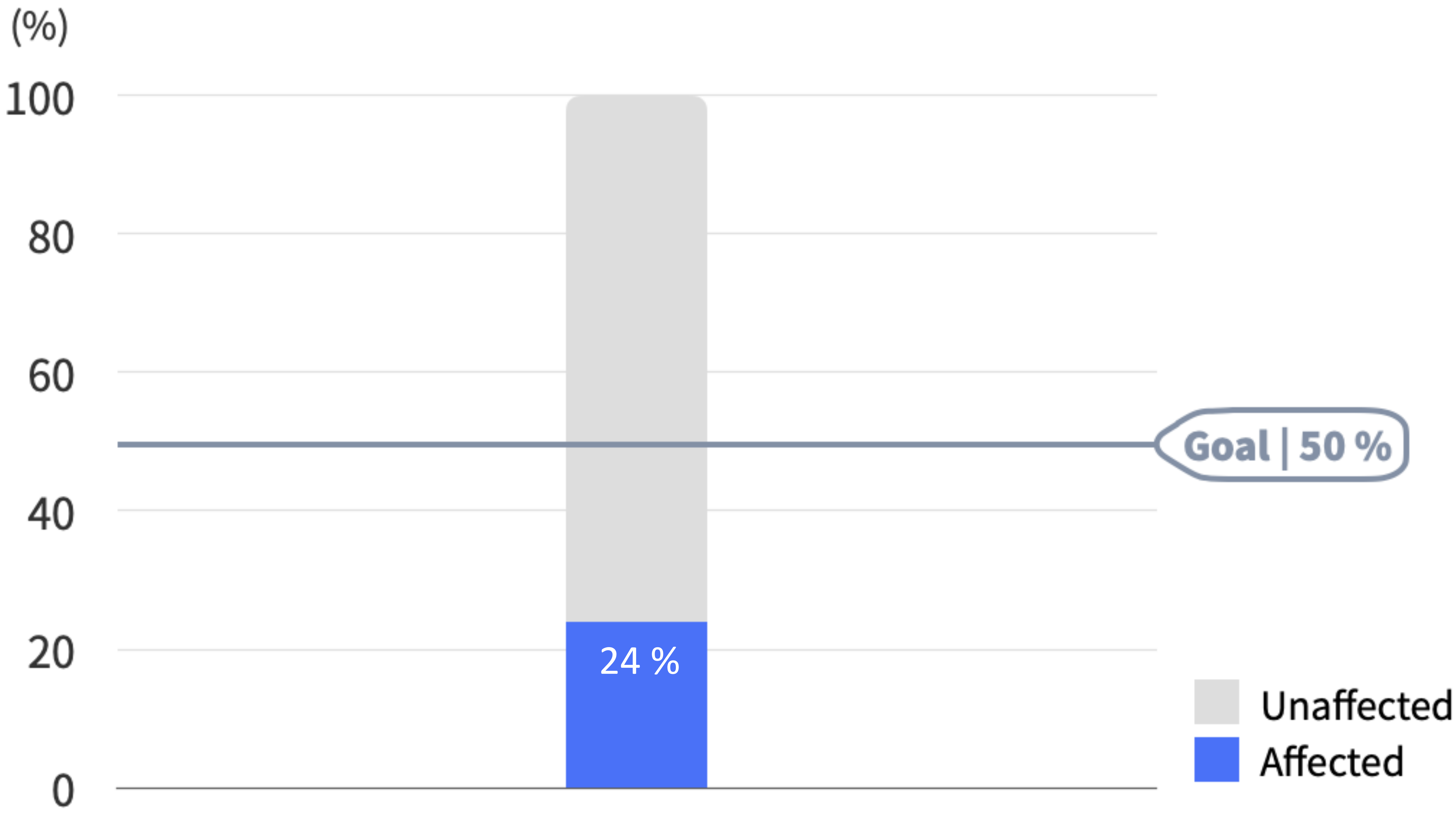

Ratio

Different Range and Granularity of Information Available

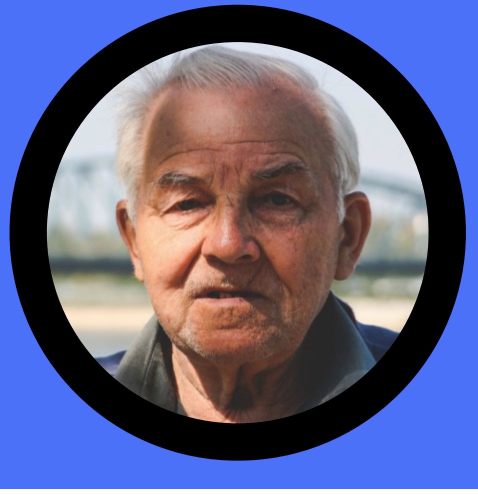

Day | Week | Month | Year

[illegible], 2020

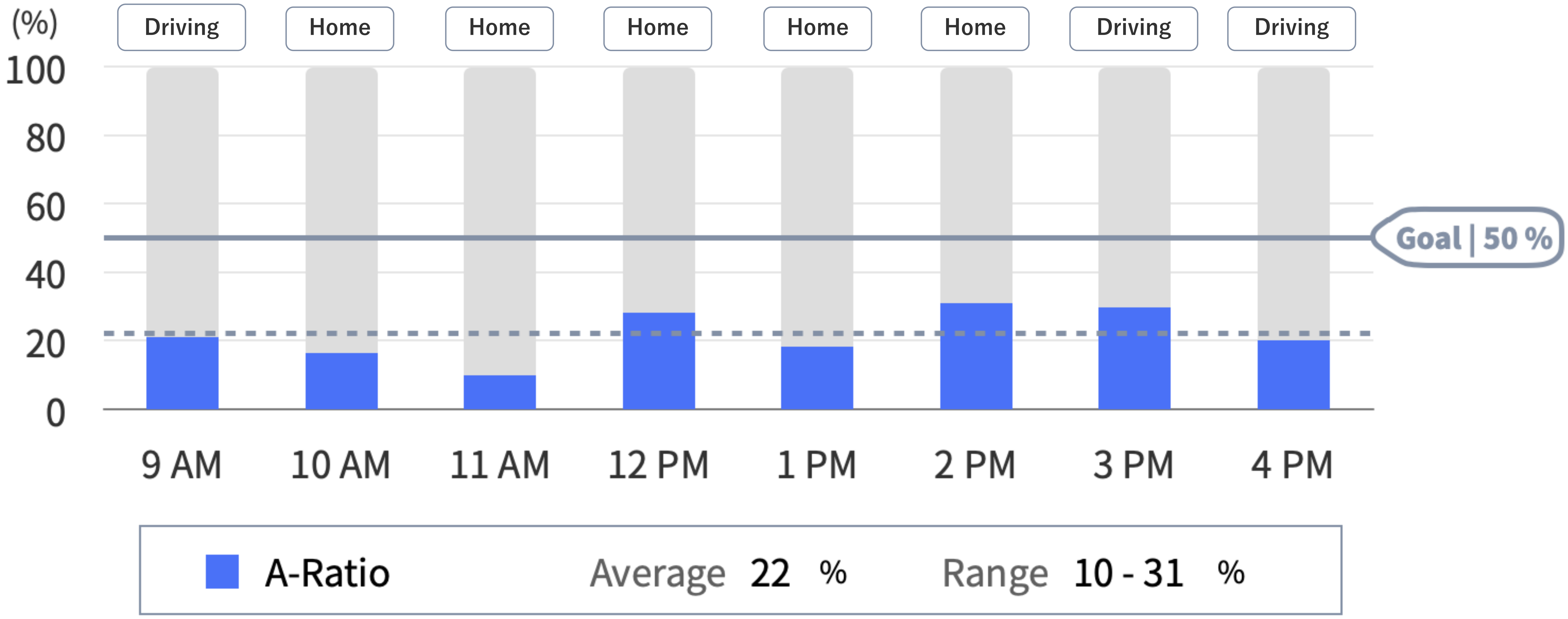

A-Duration

Duration of Affected Limb Use

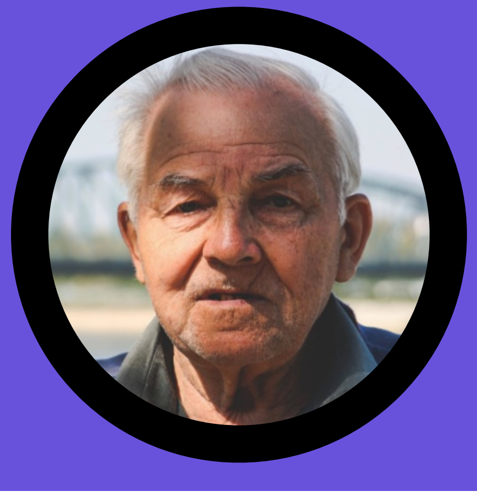

Ratio — Ratio of Affected Limb Use

A-Duration — Duration of Affected Limb Use

B-Duration — Duration of Bilateral Limb Use

A-Duration

Average A-Duration for 2 Days

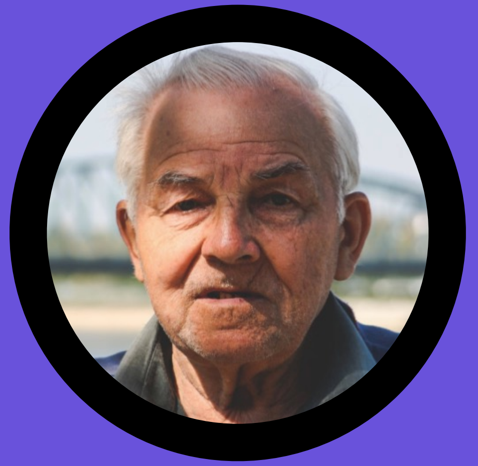

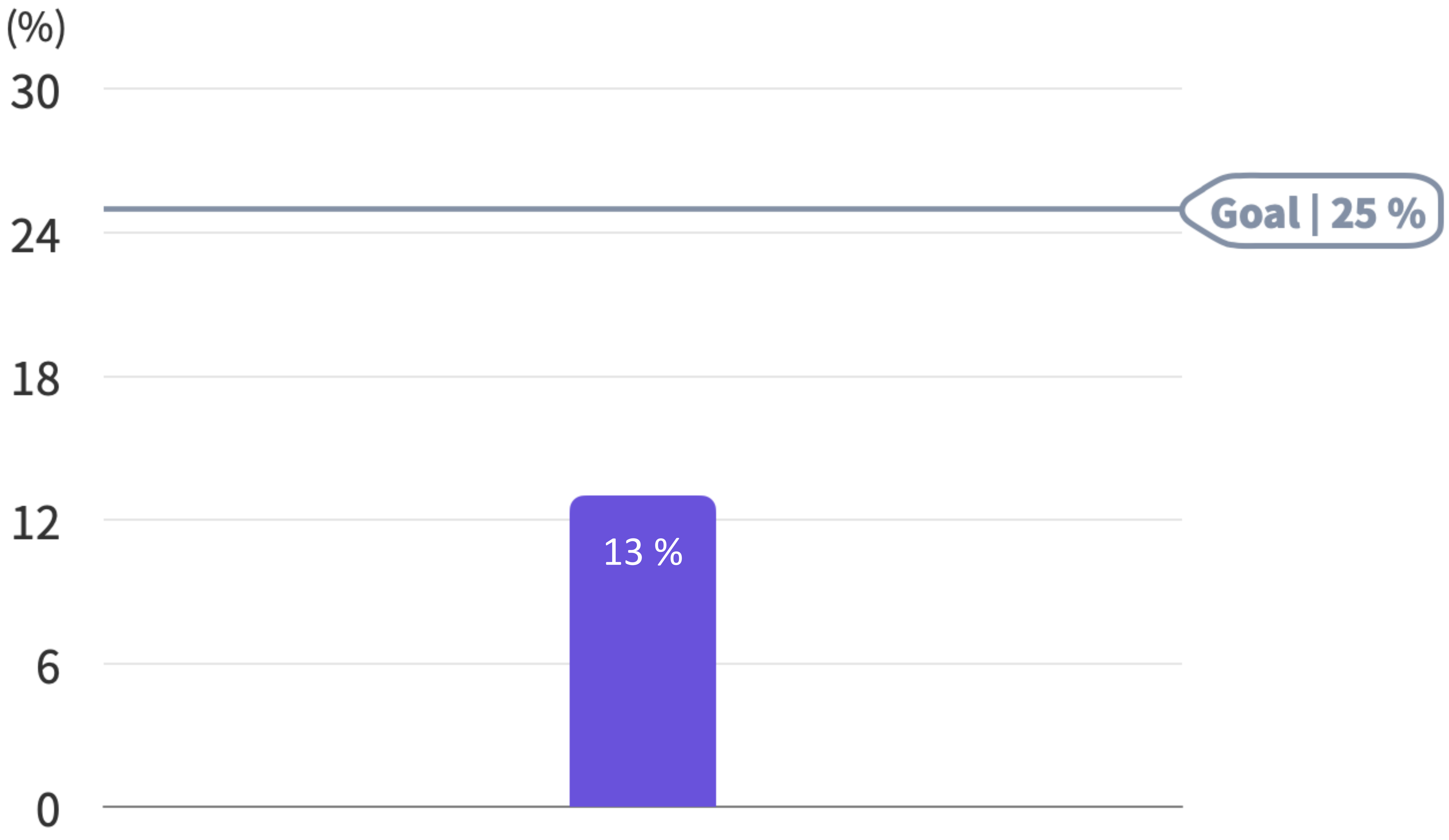

A-Duration

Different Range and Granularity of Information Available

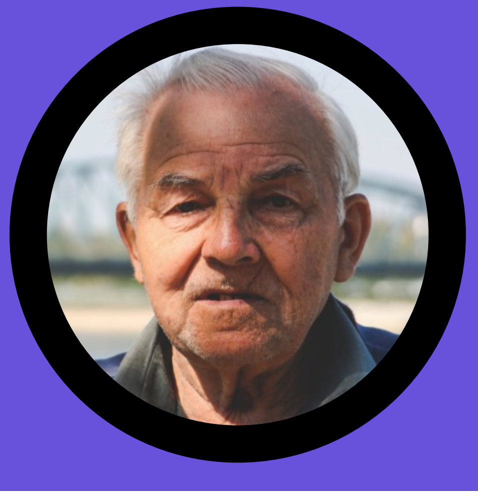

[illegible], 2020

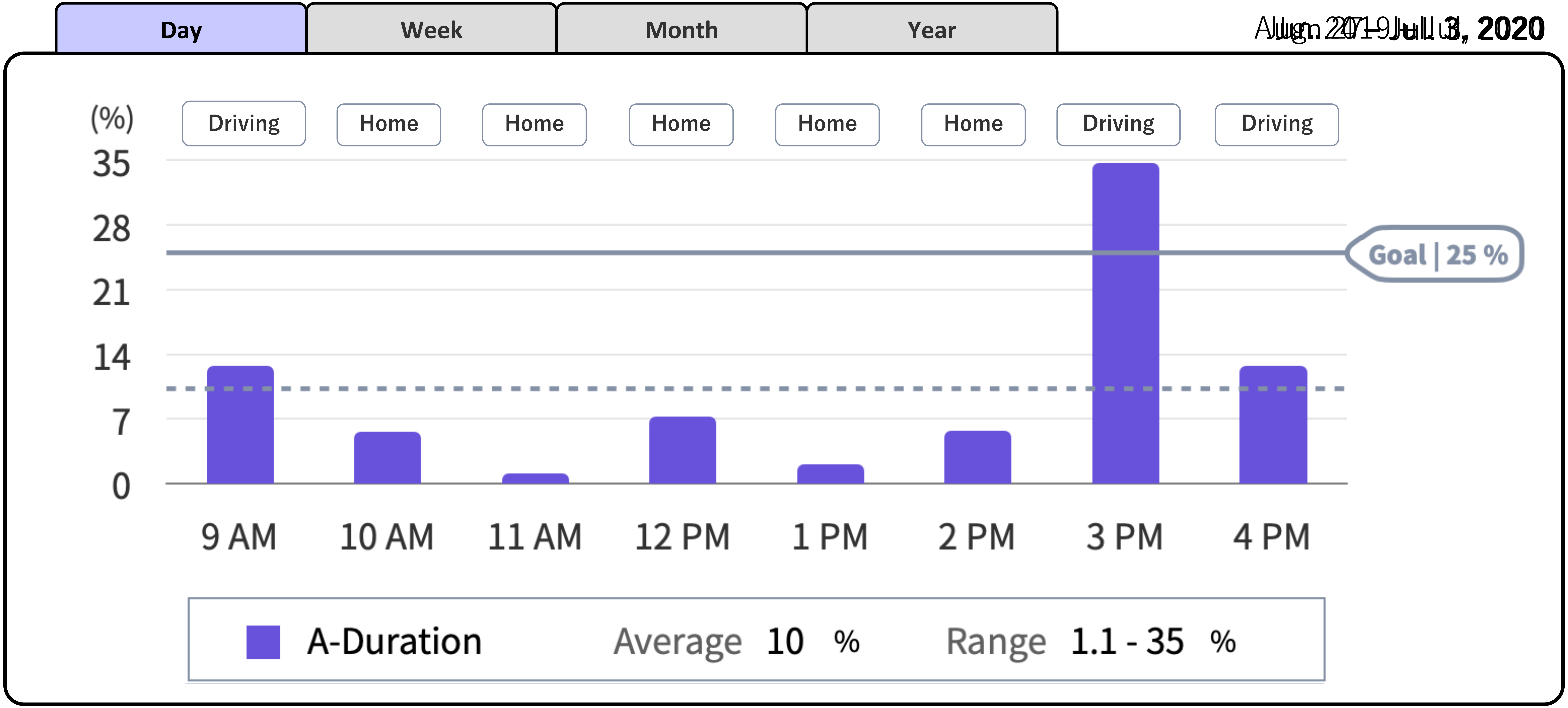

Duration of Bilateral Limb Use

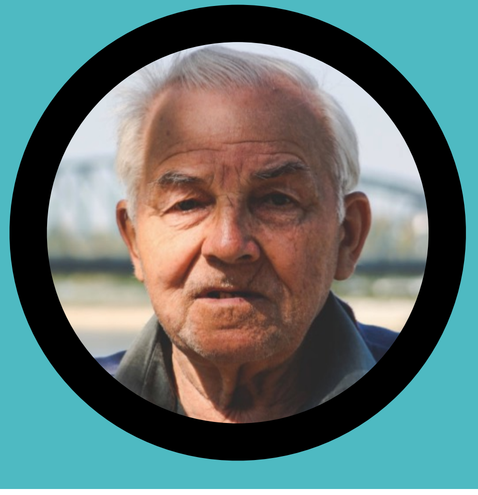

Ratio — Ratio of Affected Limb Use

A-Duration — Duration of Affected Limb Use

B-Duration — Duration of Bilateral Limb Use

B-Duration

Average B-Duration for 2 Days

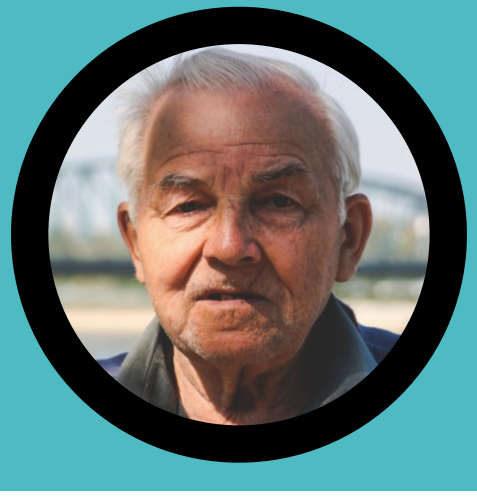

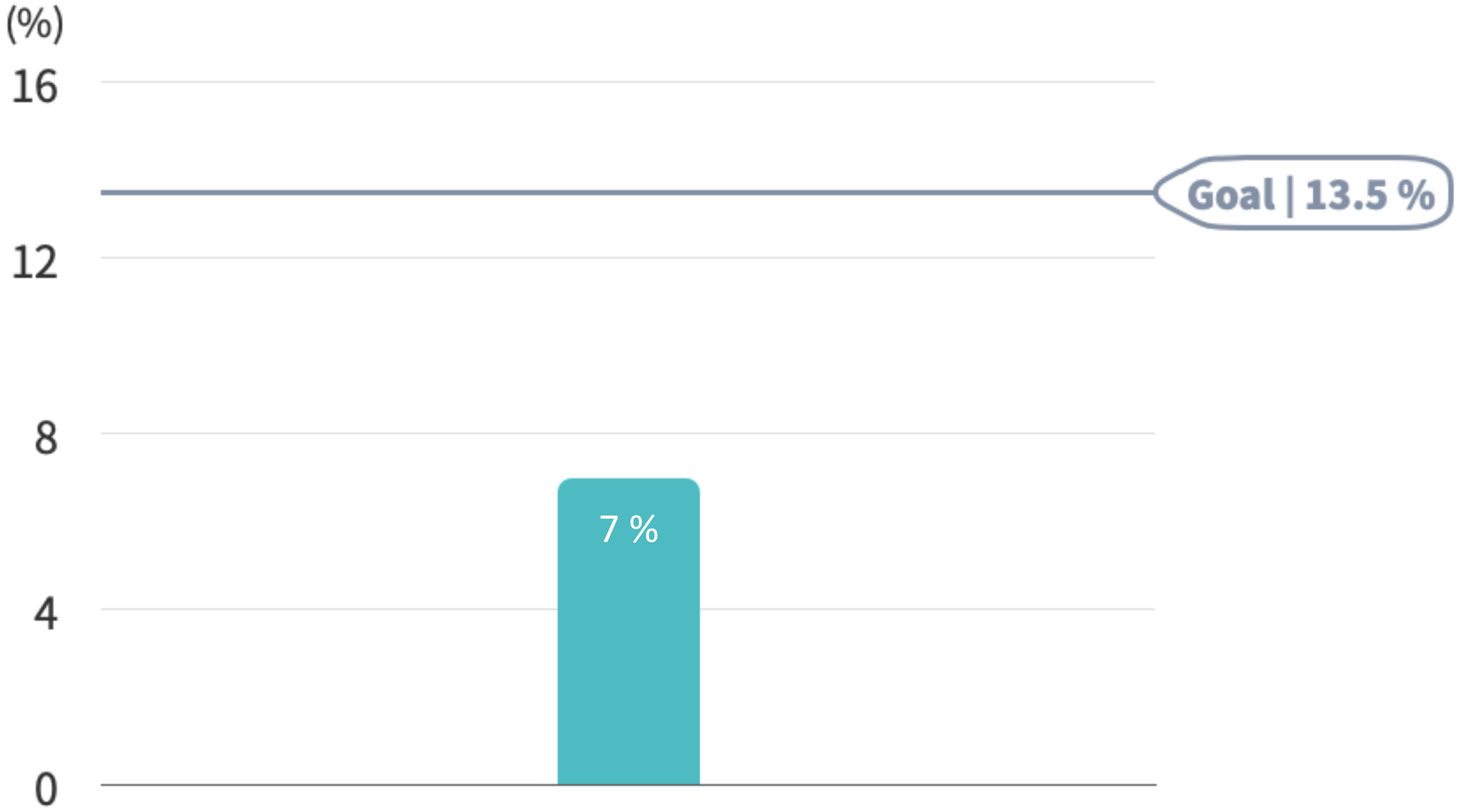

B-Duration

Different Range and Granularity of Information Available

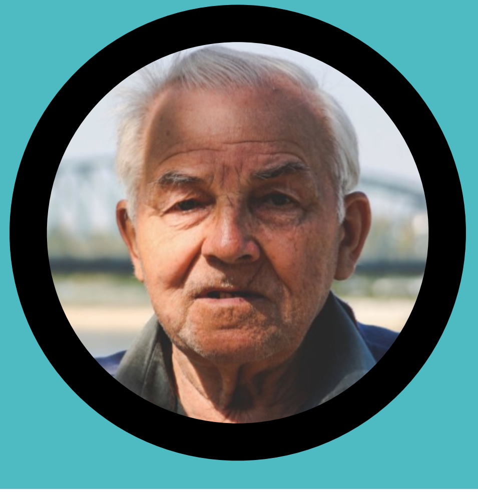

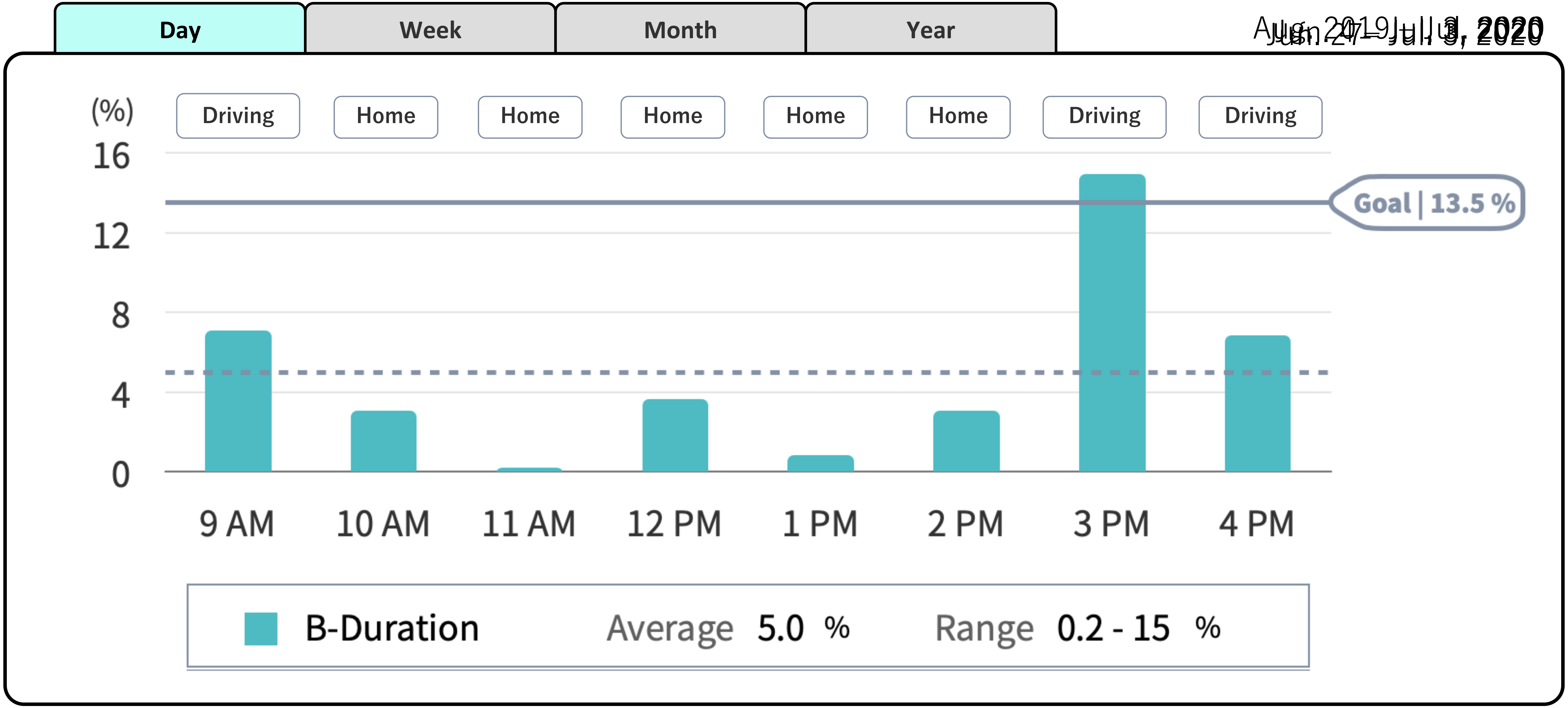

Three Different Types of Information

**Ratio**	Ratio of Affected Limb Use
**A-Duration**	Duration of Affected Limb Use
**B-Duration**	Duration of Bilateral Limb Use

Questions

How Can Sensor-based Information Change Your Practice?

A Anticipated effects to your therapy practice

B Recommended use for patients

Appendix
